# Molecular Strong Coupling and Cavity Finesse

**DOI:** 10.1021/acs.jpclett.4c00782

**Published:** 2024-07-15

**Authors:** Kishan S. Menghrajani, Adarsh B. Vasista, Wai Jue Tan, Philip A. Thomas, Felipe Herrera, William L. Barnes

**Affiliations:** † Department of Physics and Astronomy, Stocker Road, University of Exeter, Devon EX4 4QL, United Kingdom; ‡ Department of Physics, Universidad de Santiago de Chile, Av. Victor Jara 3493, Santiago 9170124, Chile; § Millennium Institute for Research in Optics, Concepción 750, Chile

## Abstract

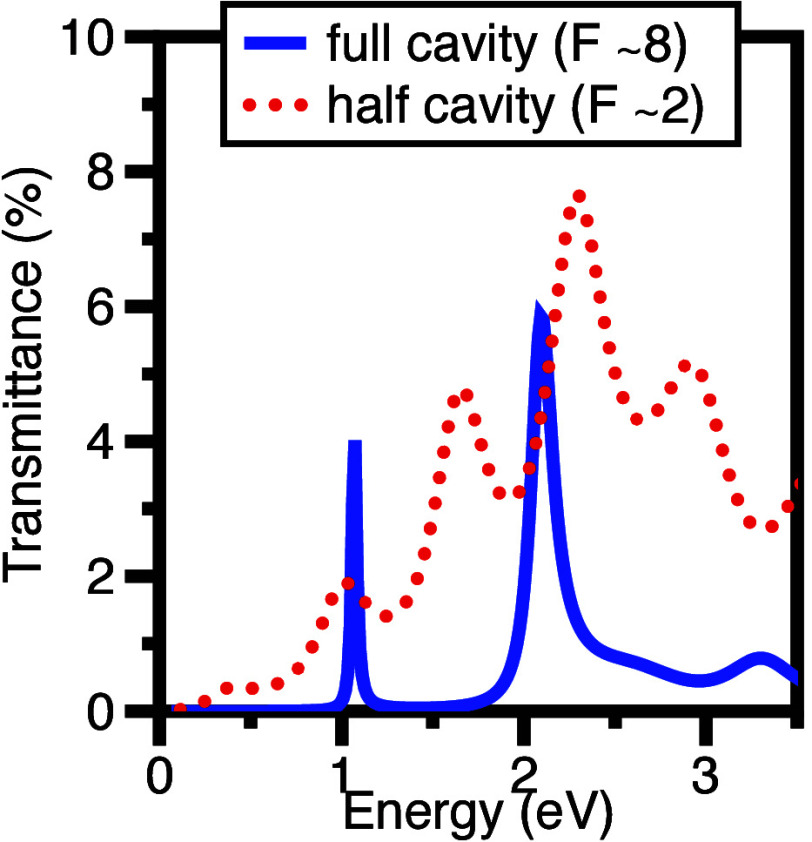

Molecular strong coupling offers exciting prospects in physics, chemistry, and materials science. While attention has been focused on developing realistic models for the molecular systems, the important role played by the entire photonic mode structure of the optical cavities has been less explored. We show that the effectiveness of molecular strong coupling may be critically dependent on cavity finesse. Specifically we only see emission associated with a dispersive lower polariton for cavities with sufficient finesse. By developing an analytical model of cavity photoluminescence in a multimode structure we clarify the role of finite-finesse in polariton formation and show that lowering the finesse reduces the extent of the mixing of light and matter in polariton states. We suggest that the detailed nature of the photonic modes supported by a cavity will be as important in developing a coherent framework for molecular strong coupling as the inclusion of realistic molecular models.

When molecules are placed inside an optical microcavity, the strong interaction between molecular resonances and cavity modes leads to the formation of hybrid states called polaritons - states that inherit characteristics of both the optical cavity modes and the molecules from which they are formed.^1,2^ This process, known as molecular strong coupling, has been extensively explored in the context of both excitonic^3−5^ and vibrational resonances.^6−8^ In the context of vibrational strong coupling there is at present much excitement owing to the prospect of modifying chemical processes,^1,2,9−13^ despite an incomplete understanding of the underlying science.^14−16^ A range of “cavity” structures have been explored, most frequently planar (Fabry–Perot) optical microcavities in which the molecules are located between two closely spaced metal or dielectric mirrors. Planar microcavities have dominated molecular strong coupling studies for many years, in both excitonic and vibrational regimes. However, such structures do not offer good access to the molecules involved, thereby limiting their applicability to cavity modified chemistry. Alternative “open” geometries have been explored, including surface plasmon modes,^17,18^ dielectric microspheres,^19^ and surface lattice resonances.^20,21^ More recently so-called “cavity-free” geometries have been investigated,^22−25^ and extensive mode splitting observed. These cavities do not use metallic or dielectric multilayer (DBR) mirrors, instead they rely on reflection from the interface of the molecular material with another dielectric to produce optical modes. While some of the reports concerning open cavities have noted changes to the molecular absorption, it remains to be seen whether such structures can be used to control chemistry effectively. Since modification of photoluminescence is a more stringent measure of strong coupling than reflectance, transmittance, absorbance and scattering,^26−29^ here we chose to explore the photoluminescence process for open, half and full cavities, in an attempt to gain better insight into cavity-free strong coupling. In doing so we identify an additional requirement that needs to be met for effective molecular strong coupling, one that highlights the vital role of cavity finesse.

In the work reported here we made use of three different planar cavity structures, shown in the top row of Figure 1: (left) an open cavity, i.e. a layer of polymer containing dye molecules supported by a silicon substrate; (center) a half-cavity, similar to (a), but with the addition of a metallic (gold) mirror between the substrate and the dye-doped polymer, and; (right) a full-cavity, similar to the half-cavity but now with a second metallic mirror added to the top of the structure. For a more extended description of the optical modes supported by the different structures see Supporting Information (SI) sections 4–6. We made use of the J-aggregated dye TDBC (5,5′,6,6′-tetrachloro-1,1′-diethyl-3,3′-di(4-sulfobutyl)-benzimidazolocarbocyanine), either dispersed in the polymer PVA, or deposited using a layer-by-layer approach.^28^ We used a silicon substrate, and made use of gold for the metallic mirrors; further details of fabrication are given in SI section 11. We measured photoluminescence and reflectance spectra as a function of polar angle, thereby enabling us to construct dispersion diagrams. We analyzed our experimental data using a transfer matrix model to calculate the reflectance, transmittance and absorption, while we made use of a coupled oscillator model to determine the modes of each system; again, details of both are given in the SI (see sections 14 and 13 respectively). To enter the strong coupling regime the collective Rabi splitting, Ω_*R*_, should be greater than the mean of the cavity and molecular spectral widths, *K* and Γ respectively (see also the note in the SI, section 1), which we can write as,^30^
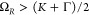
1However, as we will see below, satisfying this condition does not guarantee strong coupling as witnessed by photoluminescence; instead we find that we need to place another condition on the finesse of the cavity modes.

**Figure 1:**
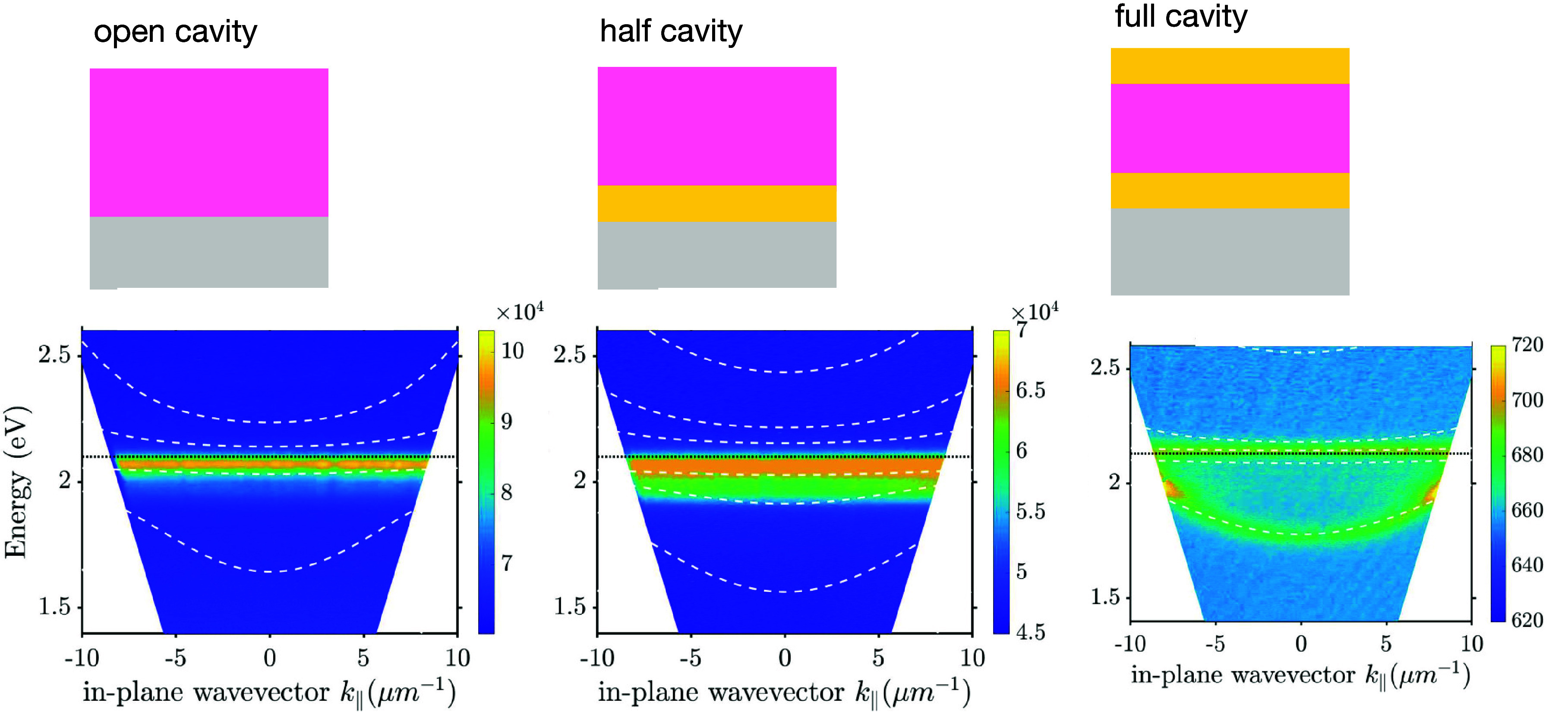
**Schematic of Cavity Structures, and measured Photoluminescence dispersion: Left: Open Cavity** consisting of a TDBC-doped polymer (PVA) film on a silicon substrate, doped-PVA thickness ∼340 nm. **Centre: Half Cavity**, as (a) but now a thin gold film is included between the substrate and the dye-doped polymer, doped-PVA thickness ∼600 nm. **Right: Full Cavity**, similar to (b) but with the addition of a top gold layer to form the second mirror of a closed cavity, the cavity thickness is ∼400 nm. Photoluminescence spectra were acquired from each sample as a function of collection angle and the data are plotted here in the form of a dispersion diagram. The white dashed lines in each PL plot show the positions of the polaritons determined from the coupled oscillator model. Further data for these samples are shown in the SI. (Note that the (nondispersive) emission in the vicinity of the molecular resonance is likely to be due to uncoupled molecules and weakly emissive dark states.).

Photoluminescence (PL) and reflectance spectra were acquired as a function of wavelength and angle. For reflectance measurements a white-light source was coupled to an objective lens (100x, 0.8 NA) and focused on to the sample. The reflected light was then collected using the same objective lens and projected onto the Fourier plane.^31^ For PL measurements, a 532 nm (green) diode-laser source was focused onto the sample and the PL was collected by the same objective lens in the backscattering configuration. Details of the optical setup are provided in section 3 of the Supporting Information.

For each system we determined the extent of the anticrossing (collective Rabi-splitting), Ω_*R*_, and the width (fwhm) of the cavity mode, *K*. The cavity mode-width was estimated from the calculated reflectance of “no-resonance” systems, where “no-resonance” means that the oscillator strength was set to zero, see for example figure S2, panel (c), and see section 7 of the Supporting Information. To estimate the collective Rabi splitting we adopted an iterative process as follows. A coupled oscillator model was used to reproduce the dispersion of the polariton modes, based on the “no-resonance” mode positions and an estimate for the Rabi frequency. The results from the coupled oscillator model were then plotted on top of the experimental (reflection and PL) data, and on top of the calculated reflection data. The value of the Rabi frequency was then adjusted to give a simultaneous best “by eye” match to the different data sets (see also section 13 of the Supporting Information). The reflectance data are thus key to determining the Rabi splitting since they exhibit both UP and LP features, however, we also ensured that the polariton positions we predict are consistent with the PL data. The full data sets are shown in figures S2–S8, a subset of the (reflectance) data are shown in Figure 2 below.

**Figure 2:**
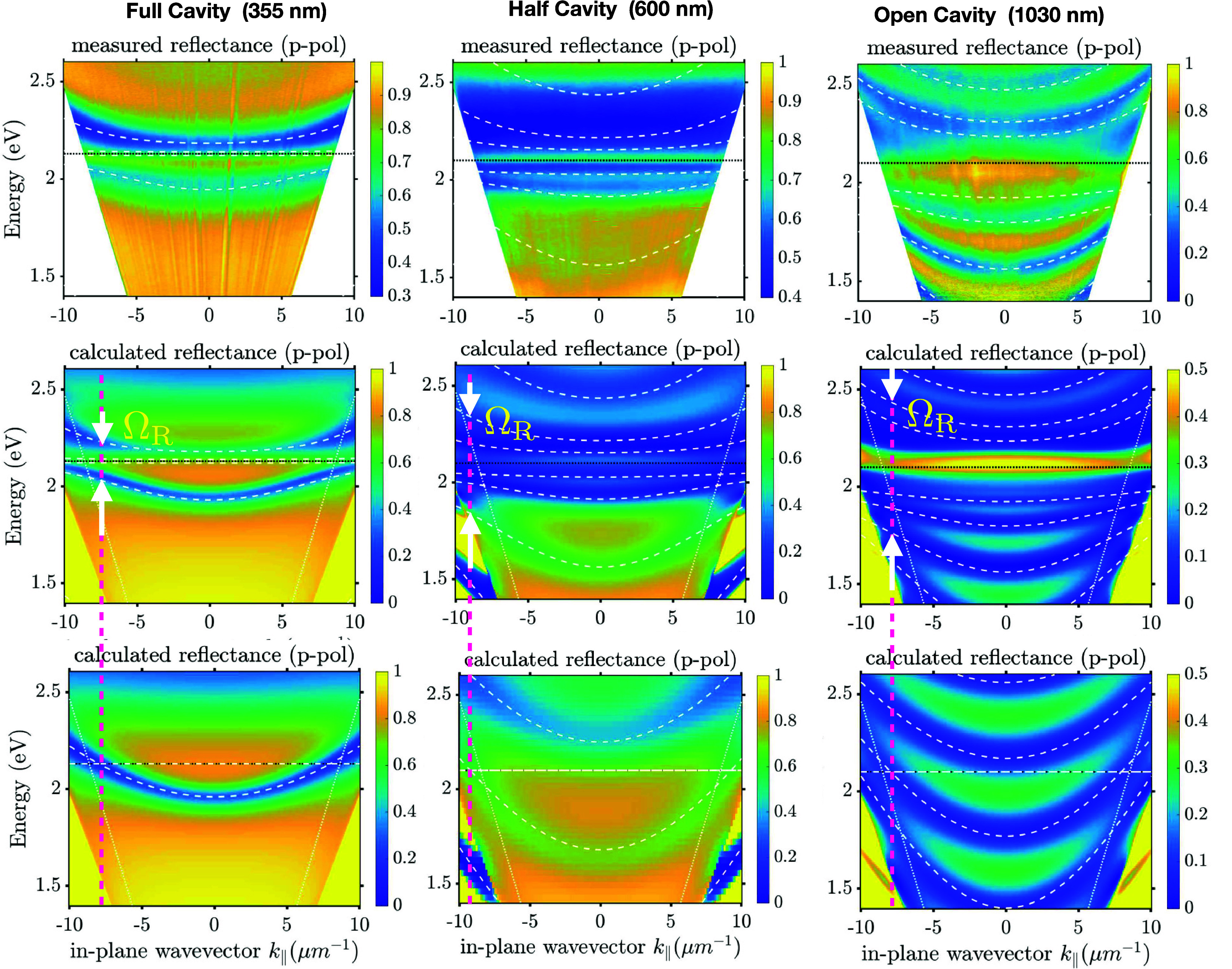
**Estimating the Rabi splitting:** In each column the top panel shows the measured reflectance, the middle column the calculated reluctance, and the lower panel the calculated reflectance with the oscillator strength set to zero. In addition, we have superimposed on each panel the results from the best “by eye” match of a coupled oscillator model to the data. The left-hand column is for a full cavity (355 nm thick), the middle column for a half-cavity (600 nm thick), the right-hand column for an open cavity (1030 nm thick). The crossing point of the key cavity mode with the excitonic resonance is shown as a magenta dotted line in each column. In each of the middle panels the arrows indicate the extent of the Rabi-splitting. Further information is given in figures S2–S8.

Let us now return to consider Figure 1 in more detail. In the second row of Figure 1, we show examples of the collected photoluminescence from the three types of cavity in the form of dispersion diagrams. Here the PL spectra have been plotted as a function of frequency and in-plane wavevector  (where λ is the wavelength of light and θ is the angle of incidence; the plane is that of the sample). Also shown as white dashed lines are the energies of the hybrid modes determined via the coupled oscillator model, further details are given in the SI. For each configuration a clear LP is present in reflectivity between 1.6 and 1.8 eV, but only in the case of the full cavity is PL associated with the LP seen. As we will show below, PL is only seen to be associated with the dispersive LP if the cavity finesse is sufficiently high.

Let us return now to discuss the PL data for each cavity configuration in turn. In the second row, left column of Figure 1 we show the measured photoluminescence dispersion. We observe a strong peak at 2.07 eV and a weaker peak at 1.97 eV. For reference a PL spectrum from a very thin (20 nm) TDBC film on Si is also shown in Supplementary Figure S9. The reference spectrum is very similar to that of the open cavity: for the open cavity case, the 2.07 eV peak is slightly broader and the 1.97 eV shoulder slightly stronger. The photoluminescence of the open cavity is also nondispersive. There is thus little if any sign that strong coupling has influenced the dye photoluminescence of this open cavity. One might argue that when the lower polariton mode at *k*_∥_ = 0 is so far in energy from the unmodified photoluminescence that no change would be expected. However, we have observed elsewhere that this need not be the case,^28^ provided there are phonon/vibrational modes that can scatter emission via the polariton. Note that there is no dispersion of the PL in the vicinity of the dispersion curve where anticrossing might be anticipated. We repeated this experiment for a thicker (∼1030 nm) TDBC film (Supplementary Figure S3) and once again found that the PL is only marginally modified (if at all) in the open cavity configuration. To investigate the absorption in the TDBC layer we made further use of transfer matrix modeling, the results are shown in figure S2 of the Supporting Information. Although there is a significant change in the absorption (in the bulk this would be a single peak) it is clear that the distortion is not due to the presence of polaritons. Instead the doublet feature in absorption in this seemingly simple sample arises from the complex interplay between absorption and the impedance that the TDBC-layer presents to incoming light.^32^ This is consistent with our previous modeling of the absorption of cavity-free strong coupling with a broad spectrum dye,^23^ which showed modified absorption but no clearly resolvable polariton modes.

The half cavity case (center column of Figure 1) appears very similar to the open cavity case (a larger PL peak at 2.07 eV and a smaller PL peak at 1.97 eV), but with a smaller difference between the magnitudes of the two peaks. Again, there is no clear mapping of the PL onto the position of the polariton modes. Calculated data for the absorption in the TDBC layer are shown in the SI, figure S4 panel (e). There is again a significant change in the absorption compared to that of the bulk, and further, compared to the case of the open cavity, there is now some indication of the absorption tracking the lower polariton mode, at least to some limited extent. Data collected from a 1630 nm thick half cavity sample (supplementary figure S5) also show a somewhat modified PL spectrum.

The measured photoluminescence dispersion for the full cavity (right-hand column of Figure 1) is significantly different from the open and half cavity cases, the PL clearly tracking the lower polariton mode. Calculated data for the absorption in the TDBC are shown in SI figure S8, panel (e). As for the PL, there is now a very significant change in the absorption that also clearly tracks the polariton modes.

It is useful at this point to compare the line spectra for the PL, for which we have chosen *k*_∥_ = 0. Figure 3 shows the PL spectra for another set of open, half and open cavities with different thicknesses than those in Figure 1, but with equivalent spectral behavior (full dispersion data in supplementary figures S3, S4, and S8). We have indicated the position of the lower polariton of the least detuned cavity mode at *k*_∥_ = 0. As a reference “uncoupled” case, a thin film of TDBC (20 nm) on Si is used, which has a strong PL peak at 2.07 eV with a weak shoulder around 1.96 eV.

**Figure 3:**
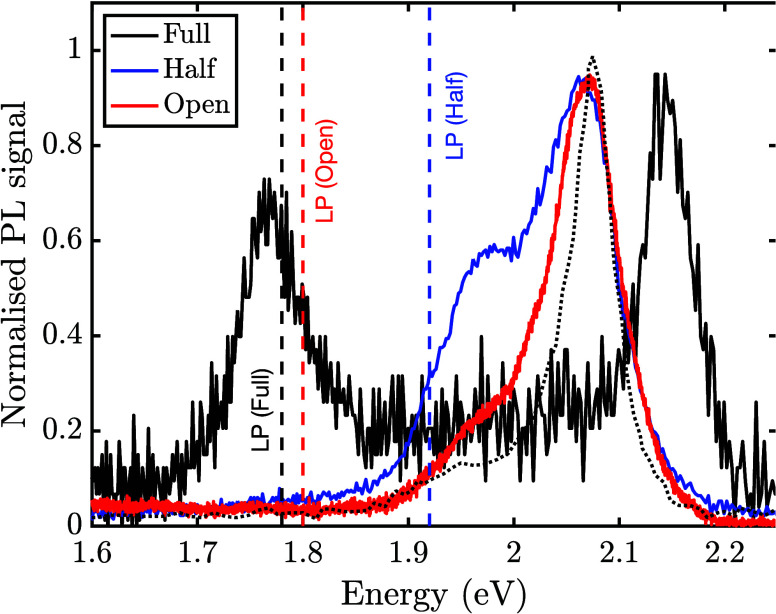
**PL line spectra**. PL for normal emission for an open cavity (1030 nm sample, red line), a half cavity (600 nm sample, blue line), and full cavity (355 nm sample, black line). The PL data have been normalized and scaled to lie between values of 0 and 1. The position of the lower polariton modes for *k*_∥_ = 0 are shown as vertical dashed lines, the lower polaritons shown here are associated with the mode that crosses the TDBC absorption energy in the zero-oscillator strength dispersion, see panels (c) in supplementary figures S3, S4, and S8. The thin film reference PL data set (dotted line) was acquired from a thin film of TDBC on a silicon substrate, see supp info section S7. Note the difference in the peak position of the “bare” PL for the full cavity data when compared to the open and half cavity data. Part of this difference can be attributed to the fact that the TDBC for this full cavity was made using the layer-by-layer technique (see supp info), while for the other two data sets the TDBC was on the polymer host PVA, again, see supp info section S7.

For the *open cavity*, the 1.97 eV PL shoulder is clearer than in the thin film and the 2.07 eV peak is broader and the PL remains nondispersive. For the *half cavity*, the 1.97 eV PL peak is substantially enhanced compared to the open cavity, but there is still no clear PL dispersion. The thicker half-cavity PL is slightly dispersive. The changes at 1.97 eV might be weakly linked to the (lower) polariton modes supported by this structure, see figure S4 (d,f). The absorption spectrum shows signs of being modified.

In contrast, for the *full cavity* both PL and absorption clearly track the lower polariton. We also note that, as commonly found,^33−35^ PL is observed from polaritons at energies lower than the molecular resonance energy, i.e. we do not see any PL associated with the upper polariton. Looking at the dispersion of the PL from the lower polariton we see that it is not uniform: PL is typically produced by the relaxation of reservoir states through the loss of vibrational energy^36^ so that PL emission is strongest when the difference between the bare molecular resonance energy (reservoir) and the polariton branch are equal to the energy of a vibrational mode.^28^

We have compared the photoluminescence from dye molecules (TDBC aggregates) located in three different cavity configurations: open, half and full cavities. In all three cases the reflectivity data indicate the strong coupling regime has been reached, see the fifth column of Table 1. The calculated absorption shows a somewhat different picture, with changes in the absorption by the TDBC for all three cavity types, but only in the case of the full cavity does the absorption track the (lower) polariton fully. We also observe changes in PL for all three samples, but again it is only for the full cavity that the PL maps onto the lower polariton. The behavior we observe in PL, Figure 3, is reminiscent of the transition from weak to strong coupling observed in reflection measurements,^37^ where an initially uncoupled peak broadens before splitting into two clearly resolvable peaks. In PL the upper polariton is not observed due to nonradiative relaxation, so instead a lower polariton branch eventually becomes distinct from the uncoupled PL peak. Ordinarily, the transition from weak to strong coupling is observed by increasing the number of molecules in a cavity, for example by using photochromic molecules.^4,37^ Here, however, similar behavior has instead been observed by modifying the cavity structure. This leads to a number of questions: how can we quantify the change in these structures that has caused this transition into the strong coupling regime, and what is the threshold for the observation of strong coupling in PL?

**Table 1:** **Spectral parameters for the different cavities**^a^

Cavity (nm)	Δω (eV) (FSR)	*K* (eV) (mode-width)	Ω_*R*_ (eV) (Rabi splitting)	2Ω_*R*_/(*K* + Γ) (>1 for SC)	*Q* (Q-factor)	**(Finesse)**
**Open**						
340	1.20 ± 0.02	0.50 ± 0.02	0.50 ± 0.03	1.75 ± 0.12	4.2 ± 0.2	**2.4** ± 0.2
1030	0.38 ± 0.01	0.17 ± 0.01	0.67 ± 0.04	5.58 ± 0.57	12.4 ± 0.7	**2.2** ± 0.2
**Half**						
600	0.64 ± 0.01	0.25 ± 0.01	0.50 ± 0.03	3.13 ± 0.27	8.4 ± 0.4	**2.6** ± 0.1
1630	0.25 ± 0.01	0.10 ± 0.01	0.35 ± 0.02	4.12 ± 0.40	21 ± 2	**2.5** ± 0.4
**Full**						
330	0.73 ± 0.01	0.13 ± 0.01	0.16 ± 0.01	1.60 ± 0.19	14 ± 4	**5.6** ± 0.6
355	0.75 ± 0.01	0.11 ± 0.01	0.20 ± 0.01	2.22 ± 0.27	19 ± 2	**6.2** ± 0.6
400	0.76 ± 0.01	0.09 ± 0.01	0.27 ± 0.02	3.38 ± 0.49	23 ± 2	8.4 ± 1.2

a The relevant figures are as follows: open cavity, figures S2 and S3; half cavities, figures S4 and S5; full cavities, S6 - S8.

Previously we have looked at whether absorption by the dye is modified in the strong coupling regime, and–as here–we found that for an open cavity there was some modification.^23^ However, that study made use of a broad spectrum dye (whereas TDBC is narrow-band–with a spectral width of Γ = 0.07 eV) – and no PL measurements were undertaken. Other work looking at strong coupling between dye molecules and particle plasmon modes found that there was clear PL arising from the lower polariton in the strong coupling regime.^27^

What are we to make of our results? To address this question we looked for a correlation between our findings and the cavity parameters, e.g. *Q*-factor. In Table 1 we have brought together the parameters for our different structures. As noted in the introduction, a conventional criterion for strong coupling is that 2Ω_*R*_ > (Γ + *K*). Based on the data in Table 1 and the fact that for TDBC, the spectral width is Γ = 0.07 eV, all of our samples meet this criterion (see fifth column of Table 1). It thus appears that this criterion is not the full story.

We next focus our attention on a previously ignored parameter, the cavity finesse, , given by  = Δω/*K*, where Δω is the free spectral range (FSR), i.e. the frequency separation of adjacent modes, see also SI section 7. Looking now at the penultimate column in Table 1, there appears to be a clear correlation between the behavior we see in our PL results and the cavity finesse. We find that for low finesse structures,  ∼ 2, the PL does not track the (reflectance) lower polariton, even when the Rabi splitting exceeds the line widths. For higher finesse values,  > 4, the PL does track the (reflectance) lower polariton.

Based on our analysis of the cavity finesse (see Table 1) we suggest an additional criterion, to supplement the usual strong coupling criterion, (1), we suggest,
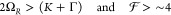
2We have plotted our data in this form in Figure 4; solid and gray lines indicate the two criteria. For the data associated with the cavities that we have investigated here (data points with red error bars), we see that the open and half cavities are all such that the free spectral range is too low. In contrast, the values for the three full cavities have both sufficient finesse ***and*** sufficient coupling strength for effective strong coupling.

**Figure 4:**
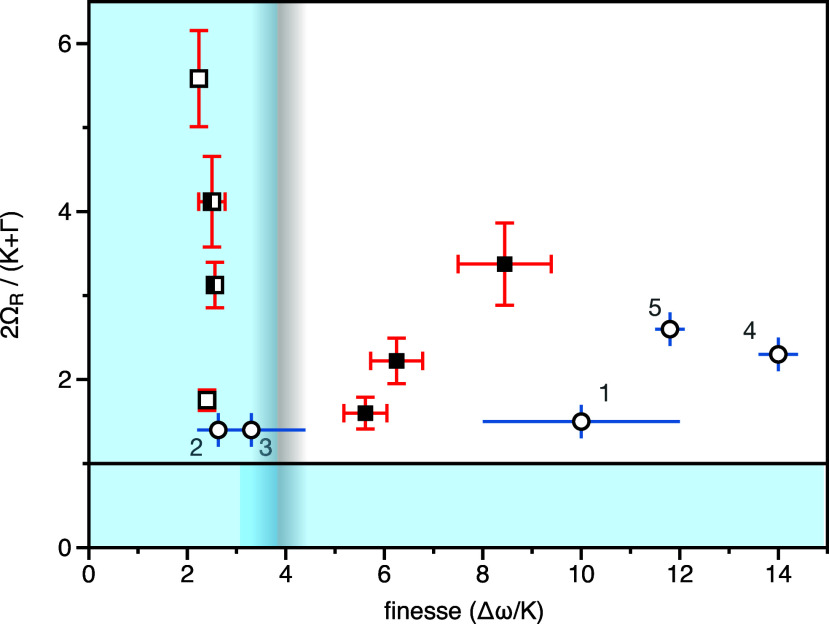
**Strong coupling parameter space**. Values of 2Ω_*R*_/(*K* + Γ) and Δω/*K* (finesse) for each of the cavities we investigated. The error bars (red) are derived from the data in Table 1. Data from open cavities are indicated by an open square, from half cavities by a half-filled square, and from full cavities by a filled square. Also shown are data points (together with associated error bars, in this case blue) based on other reports, for details see main text. The horizontal line at 2Ω_*R*_/(*K* + Γ) = 1 indicates the “usual” strong coupling condition. The vertical blurred line indicates our suggested criterion based on cavity finesse. Our 2D criterion is satisfied in the region of white background.

To explore these ideas further we also plot in Figure 4 data extracted from a number of reports in the literature. Point 1 is for the dye-coated plasmonic nanoprism reported by Wersall et al.^26^ It is clear that for the plasmonic particle system investigated by Wersall et al. effective strong coupling was achieved, and their PL data confirm this. Point 2 is for the open dielectric cavity of Thomas et al.^23^ As for the open cavities we have explored here, it is perhaps marginal whether this system has attained the effective strong coupling regime. In this case, coupling to a higher-order electromagnetic mode would greatly lower *K*, potentially pushing this system into the effective strong coupling regime. Point 3 is for the dielectric microsphere of Vasista et al.^19^ It is clear in this case that while the coupling strength is adequate, the finesse is too low. It may be that a reduction in microsphere size (resulting in an increase in Δω) would allow the effective strong coupling regime to be reached. Points 4 and 5 are planar Fabry–Perot cavities used in two studies that report modifications to chemical reactions due to vibrational strong coupling. Point 4 corresponds to the work of Thomas et al.,^12^ while point 5 corresponds to the work of Ahn et al.^13^ In both cases the effective strong coupling regime is comfortably reached. More information on these data is given in the SI.

How might we understand this requirement of a lower limit on the finesse in multimode cavities for effective strong coupling? Molecular strong coupling relies on the coherent exchange of energy between a set of molecular resonators and a cavity mode. If the finesse is too low then the molecular resonators can interact with multiple cavity modes simultaneously, thus changing the properties of the lower and upper polariton states around the molecular resonance. In Figure 5 we schematically compare the type of mode mixing present in high and low finesse cavities. Three photonic modes {*C*_–1_, *C*, *C*_+1_} are shown in order of increasing energy, interacting with an excitonic mode (*X*) that is resonant with the central mode *C*. For high finesse, only the *C* and *X* modes exchange energy, giving the usual single-mode picture of light-matter interaction. In low finesse cavities, the exciton content is shared among multiple partially overlapping cavity modes, which changes the emission properties of the lower polariton in the central spectral region.

**Figure 5:**
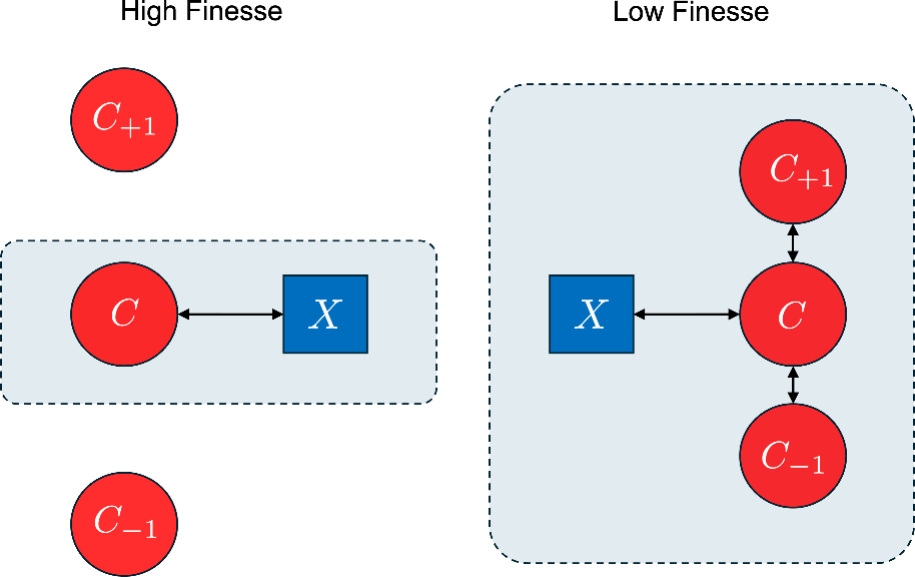
**Schematic of effect of multiple photonic modes**. The schematic shows three photonic modes {*C*_–1_, *C*, *C*_+1_} coupling to a single molecular resonance. For high finesse cavities (left) the dominant interaction is between the molecular exciton mode (*X*) and the energetically closest photonic mode (*C*). In low-finesse cavities (right), the exciton content is shared between neighboring partially overlapping cavity modes, thus modifying the character of polariton emission signals.

The consequences of this multimode mixing process in cavity PL can be understood by breaking down the cavity emission into a multistep process in which molecular dipoles are first pumped incoherently from the ground state *S*_0_ to the lowest excited state *S*_1_, via UV excitation *S*_0_ → *S*_*n*_ followed by ultrafast radiationless relaxation *S*_*n*_ → *S*_1_,^38^ they then give up their excitation energy to the vacuum cavity field creating individual confined photons, which finally decay to the far field through the mirrors at rate *K/2*, thereby generating the PL signal.

To model PL in a multimode cavity with tunable finesse, we extend the theoretical analysis of Herrera and Spano,^39,40^ by explicitly modeling the probability of exciting molecular dipoles pumped incoherently at rate *W* and including multiple cavity modes at discrete frequencies *ω*_*q*_ (*q* an integer). Assuming that the coupled light-matter state is such that no coherence between polaritonic eigenstates is present and depletion of the ground state due to incoherent pumping is negligible, the stationary PL spectrum is then given by,
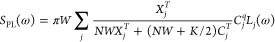
3where the discrete index *j* labels each polaritonic eigenstate in the single excitation manifold (including dark states^39^), *X*_*j*_^*T*^ is the total exciton content of the *j*-th eigenstate, *C*_*j*_^*q*^ is the photon content of the *j*-th state in the *q*-th cavity mode and *C*_*j*_^*T*^ = ∑_*q*_*C*_*j*_^*q*^ is the total photon content summed over all cavity modes. *L*_*j*_(ω) is a normalized Lorentzian response function with central eigenfrequencies *ω*_*j*_ and bandwidth Γ_*j*_ (fwhm), which for simplicity we set to Γ_*j*_ = *K* for all states. *N* is the number of molecular dipoles. The derivation of eq 3 is given in Section 10 of the Supporting Information.

At this point it is worth looking at the structure of eq 3, specifically the physical significance of the factors involved. The quantity we are calculating is the strength of the PL signal, *S*_*PL*_. First, for low pumping rates (no saturation) the strength of the PL signal is directly proportional to the pumping rate *W*. Second, the factor *X*_*j*_^*T*^/[*NWX*_*j*_^*T*^ + (*NW* + *K*/2)*C*_*j*_^*T*^] gives the probability that the lower polariton state is occupied after incoherent pumping of the dipoles. Third, the factor *C*_*j*_^*q*^ gives the photon content of the photon content of the *j*-th polariton state, it is this “fraction” of the state that is available to yield photons that leak out of the cavity sample. Finally, as noted above, *L*_*j*_(ω) is a (Lorentzian) line shape function.

Figure 6(a) shows the calculated spectrum of an idealized two-mode cavity with tunable finesse. The central mode *q* = *m* is kept at exact resonance with a molecular transition at 2.1 eV, and the energy separation Δ the lower *q* = *m*–1 mode is varied. The positions of the LP, UP and exciton lines are marked. An ensemble of *N* molecular dipoles is equally coupled to both cavity modes with local coupling strength  (eV), which gives a Rabi spliting of Ω_*R*_ = 0.4 eV for large Δ. We choose this coupling magnitude such that the usual single-mode picture of strong coupling holds for the resonant mode (*K* = 0.2 eV, Γ = 0.05 eV).

**Figure 6:**
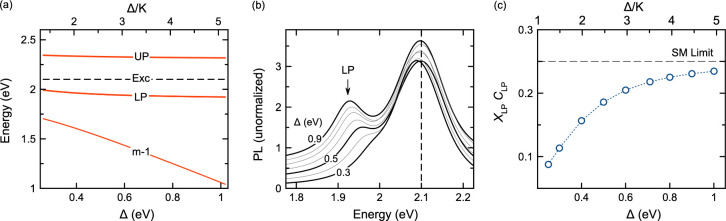
**PL in a two-mode cavity**. (a) Calculated polariton spectrum of a two-mode cavity as a function of the mode energy separation Δ. The central *q* = *m* mode strongly couples to the molecular resonance at *ω*_*e*_ = 2.1 eV and is kept fixed in energy. The lower *q* = *m*–1 mode approaches the molecular resonance with decreasing Δ. (b) Simulated PL spectra of the two-mode cavity in panel (a), for an ensemble of *N* in-homogeneously broadened molecular dipoles centered at 2.1 eV (vertical dashed line, σ = 0.021 eV). Curves are labeled by the value of Δ. LP denotes lower polariton. (c) Calculated PL spectral weight *X*_LP_*C*_LP_ at the LP energy as a function of Δ, for the same parameters in panel (b). The single-mode limit *X*_LP_*C*_LP_ = 1/4 is marked with a dashed horizontal line. (Note that the spectral weight product *X*_LP_*C*_LP_ comes from eq 3 where here we have set *j* = LP, *q* = *m*, and we have dropped the other indices for notational simplicity.) In all cases we set *N* = 150,  eV, *K* = 0.2 eV, and *NW*/*K* = 10^–4^. Curves are averaged over 650 disorder configurations. In panels (a) and (c) the finesse (Δ/*K*) is plotted on the upper abcissa.

Figure 6(b) shows the PL signal at the LP energy, calculated using eq 3, for different mode separation energies Δ, (Δ *i*s proportional to finesse). A Gaussian distribution of molecular transition frequencies is assumed (*ω̅*_*e*_ = 2.1 eV, σ = 21 meV, fwhm = 0.05 eV). As the finesse changes, the central emission feature near the bare molecular resonance remains largely unaltered, in qualitative agreement with the experimental comparison in Figure 3, however the PL from the lower polariton changes substantially. The lower polariton feature in PL emerges with increasing intermode separation Δ, as controlled by the spectral weight product *X*_LP_*C*_LP_ in eq 3 (where we have set *j* = LP, *q* = *m*, and we drop the other indices for notational simplicity). This product can be understood as the degree of admixing between light and matter at the LP energy. When a light-matter eigenstate at frequency *ω*_*j*_ is either purely photonic (*X*_*j*_^*T*^ = 0) or purely excitonic (C_*j*_^*q*^ = 0), the PL signal is strongly suppressed.

For a single-mode resonant cavity with a spectrally homogeneous ensemble of molecules (σ = 0), the lower polariton state in strong coupling has *X*_LP_ ≈ 1/2 and *C*_LP_ ≈ 1/2, which sets the optimal mixing limit for the PL spectral weight *C*_LP_*X*_LP_ ≈ 1/4. Figure 6(c) shows how the PL spectral weight of the LP state monotonically decreases from this asymptotic upper limit as the energy separation between modes decreases. In addition to the change in spectral weight, for intermode energy separation Δ *c*omparable or smaller than the collective Rabi coupling , we also expect level pushing of the LP from below toward the molecular resonance, for constant light-matter coupling strength. The combination of reduced spectral weight and level pushing gives the spectral progression shown in Figure 6(b).

In summary, we investigated the photoluminescence, reflectance, and absorption associated with a range of dye-doped cavities. While in all the configurations we studied we saw evidence of strong coupling in the reflectance data, this was not true for PL. We only saw PL associated with a dispersive LP for the full cavities. For the other cavity configurations that we examined we saw nondispersive emission in the vicinity of the excitonic resonance. While a full analysis of the details of this nondispersive emission is beyond the scope of the present study, it is likely to include emission from uncoupled molecules (aggregates) and weakly emissive dark states. We compared a variety of spectral parameters with the behavior we observed in photoluminescence, and found a correlation with cavity finesse. We developed a theoretical model of photoluminescence under strong coupling so as to specifically include coupling between adjacent photonic modes, coupling that arises when the photonic modes are of low finesse. Our model provides a conceptually straightforward explanation of our results; the dominant effect of this coupling in low finesse situations is that coupling between adjacent photonic modes reduces the matter content of the polariton, thus reducing the probability of polariton emission. The absence of PL associated with the dispersive LP in low finesse situations is thus a result of the reduced (excitonic) content of the LP due to “sharing” of the exciton content among more than one cavity mode. While our aim was to better understand strong coupling in open cavities, we have arrived at a more general conclusion about effective strong coupling: that in addition to the usual condition on the coupling strength, an additional condition based on the cavity finesse needs to be met. This is perhaps not so surprising. The “traditional” strong coupling criterion is based on considering a single molecular resonance interacting with single cavity mode. A natural consequence is that the hybrid polariton modes that arise are half matter (exciton), half light (cavity mode). When more than one cavity mode is involved things can become more complicated and this simple 50/50 light-matter distribution no longer applies.^41^ As we have shown here, when the finesse is low then neighboring cavity modes can interact, leading to a reduction in the exciton content of a given polariton mode, see Figure 5. We suggest that in low finesse situations the “traditional” strong coupling criterion no longer ensures sufficient mode-mixing for all processes, e.g. photoluminescence, to be tied to the polariton modes. We expect the influence of finesse on strong coupling to apply to other optical microcavities, plasmonic nanocavities etc., as well as infrared resonators. Future experiments using novel molecular cavity designs, as well as realistic microscopic quantum theory that includes the entire cavity mode profile, molecular dephasing and collective relaxation effects, will further refine our fundamental understanding of molecular strong coupling.

## Data Availability

Data associated with these results can be found at https://doi.org/10.24378/exe.5306.

## Supporting Information Available

Strong coupling criteria; Optical modes of different cavity structures; Optical Fourier setup; Open Cavities; Half Cavities; Full Cavities; Estimating the Free Spectral Range and the cavity mode line width; Photoluminescence from different thin TDBC layers, Literature Values; Microcavity PL with finite finesse; Sample preparation; Reflectance measurements; coupled oscillator models; transfer matrix modeling; Technical details re: TOC figure (PDF)

## References

[ref1] Ebbesen T. W. Hybrid Light–Matter States in a Molecular and Material Science Perspective. Acc. Chem. Res. 2016, 49, 2403–2412. 10.1021/acs.accounts.6b00295. 27779846

[ref2] Herrera F.; Owrutsky J. Molecular polaritons for controlling chemistry with quantum optics. J. Chem. Phys. 2020, 152, 100902 10.1063/1.5136320. 32171209

[ref3] Lidzey D. G.; Bradley D. D. C.; Skolnick M. S.; Virgili T.; Walker S.; Whittaker D. M. Strong exciton-photon coupling in an organic semiconductor microcavity. Nature 1998, 395, 53–55. 10.1038/25692.

[ref4] Schwartz T.; Hutchison J. A.; Genet C.; Ebbesen T. W. Reversible Switching of Ultrastrong Light-Molecule Coupling. Phys. Rev. Lett. 2011, 106, 196405 10.1103/PhysRevLett.106.196405. 21668181

[ref5] Polak D.; et al. Manipulating molecules with strong coupling: harvesting triplet excitons in organic exciton microcavities. Chem. Sci. 2020, 11, 343–354. 10.1039/C9SC04950A. 32190258 PMC7067247

[ref6] Shalabney A.; George J.; Hutchison J.; Pupillo G.; Genet C.; Ebbesen T. W. Coherent coupling of molecular resonators with a microcavity mode. Nat. Commun. 2015, 6, 1–6. 10.1038/ncomms6981. PMC4308833 25583259

[ref7] Long J. P.; Simpkins B. S. Coherent coupling between a molecular vibration and Fabry-Perot optical cavity to give hybridised states in the strong coupling limit. ACS Photonics 2015, 2, 130–136. 10.1021/ph5003347.

[ref8] Takele W. M.; Piatkowski L.; Wackenhut F.; Gawinkowski S.; Meixner A. J.; Waluk J. Scouting for strong light–matter coupling signatures in Raman spectra. Phys. Chem. Chem. Phys. 2021, 23, 16837–16846. 10.1039/D1CP01863A. 34323915

[ref9] Yuen-Zhou J.; Menon V. M. Polariton chemistry: Thinking inside the (photon) box. Proc. Natl. Acad. Sci. U. S. A. 2019, 116, 5214–5216. 10.1073/pnas.1900795116. 30858328 PMC6431159

[ref10] Garcia-Vidal F. J.; Ciuti C.; Ebbesen T. W. Manipulating matter by strong coupling to vacuum fields. Science 2021, 373, eabd0336 10.1126/science.abd0336. 34244383

[ref11] Hirai K.; Hutchison J. A.; Uji-i H. Recent Progress in Vibropolaritonic Chemistry. ChemPlusChem. 2020, 85, 1981–1988. 10.1002/cplu.202000411. 32869494

[ref12] Thomas A.; Lethuillier-Karl L.; Nagarajan K.; Vergauwe R. M. A.; George J.; Chervy T.; Shalabney A.; Devaux E.; Genet C.; Moran J.; Ebbesen T. W. Tilting a ground-state reactivity landscape by vibrational strong coupling. Science 2019, 363, 615–619. 10.1126/science.aau7742. 30733414

[ref13] Ahn W.; Triana J. F.; Recabal F.; Herrera F.; Simpkins B. S. Modification of ground-state chemical reactivity via light-matter coherence in infrared cavities. Science 2023, 380, 1165–1168. 10.1126/science.ade7147. 37319215

[ref14] Vurgaftman I.; Simpkins B. S.; Dunkelberger A. D.; Owrutsky J. C. Negligible Effect of Vibrational Polaritons on Chemical Reaction Rates via the Density of States Pathway. J. Phys. Chem. Lett. 2020, 11, 3557–3562. 10.1021/acs.jpclett.0c00841. 32298585

[ref15] Imperatore M. V.; Asbury J. B.; Giebink N. C. Reproducibility of cavity-enhanced chemical reaction rates in the vibrational strong coupling regime. J. Chem. Phys. 2021, 154, 191103 10.1063/5.0046307. 34240900

[ref16] Vurgaftman I.; Simpkins B. S.; Dunkelberger A. D.; Owrutsky J. C. Comparative analysis of polaritons in bulk, dielectric slabs, and planar cavities with implications for cavity-modified reactivity. J. Chem. Phys. 2022, 156, 034110 10.1063/5.0078148. 35065567

[ref17] Baieva S.; Ihalainen J. A.; Toppari J. J. Strong coupling between surface plasmon polaritons and β-carotene in nanolayered system. J. Chem. Phys. 2013, 138, 044707 10.1063/1.4776233. 23387615

[ref18] Törmä P.; Barnes W. L. Strong coupling between surface plasmon polaritons and emitters: a review. Rep. Prog. Phys. 2015, 78, 013901 10.1088/0034-4885/78/1/013901. 25536670

[ref19] Vasista A.; Barnes W. L. Molecular monolayer strong coupling in dielectric soft microcavities. Nano Lett. 2020, 20, 1766–1773. 10.1021/acs.nanolett.9b04996. 32069420 PMC7581308

[ref20] Yadav R. K.; Otten M.; Wang W.; Cortes C. L.; Gosztola D. J.; Wiederrecht G. P.; Gray S. K.; Odom T. W.; Basu J. K. Strongly Coupled Exciton–Surface Lattice Resonances Engineer Long-Range Energy Propagation. Nano Lett. 2020, 20, 5043–5049. 10.1021/acs.nanolett.0c01236. 32470309

[ref21] Verdelli F.; Schulpen J. J. P. M.; Baldi A.; Rivas J. G. Chasing Vibro-Polariton Fingerprints in Infrared and Raman Spectra Using Surface Lattice Resonances on Extended Metasurfaces. J. Phys. Chem. C 2022, 126, 7143–7151. 10.1021/acs.jpcc.2c00779. PMC9059191 35521632

[ref22] Georgiou K.; Jayaprakash R.; Lidzey D. G. Strong Coupling of Organic Dyes Located at the Surface of a Dielectric Slab Microcavity. J. Phys. Chem. Lett. 2020, 11, 9893–9900. 10.1021/acs.jpclett.0c02751. 33170714

[ref23] Thomas P. A.; Menghrajani K. S.; Barnes W. L. Cavity-Free Ultrastrong Light-Matter Coupling. J. Phys. Chem. Lett. 2021, 12, 6914–6918. 10.1021/acs.jpclett.1c01695. 34280306 PMC8327311

[ref24] Canales A.; Baranov D. G.; Antosiewicz T. J.; Shegai T. Abundance of cavity-free polaritonic states in resonant materials and nanostructures. J. Chem. Phys. 2021, 154, 024701 10.1063/5.0033352. 33445887

[ref25] Georgiou K.; Athanasiou M.; Jayaprakash R.; Lidzey D. G.; Itskos G.; Othonos A. Strong coupling in mechanically flexible free-standing organic membranes. J. Chem. Phys. 2023, 159, 234303 10.1063/5.0178144. 38112504

[ref26] Wersäll M.; Cuadra J.; Antosiewicz T. J.; Balci S.; Shegai T. Observation of Mode Splitting in Photoluminescence of Individual Plasmonic Nanoparticles Strongly Coupled to Molecular Excitons. Nano Lett. 2017, 17, 551–558. 10.1021/acs.nanolett.6b04659. 28005384

[ref27] Wersäll M.; Munkhbat B.; Baranov D. G.; Herrera F.; Cao J.; Antosiewicz T. J.; Shegai T. Correlative Dark-Field and Photoluminescence Spectroscopy of Individual Plasmon–Molecule Hybrid Nanostructures in a Strong Coupling Regime. ACS Photonics 2019, 6, 2570–2576. 10.1021/acsphotonics.9b01079.

[ref28] Vasista A. B.; Menghrajani K. S.; Barnes W. L. Polariton assisted photoemission from a layered molecular material: role of vibrational states and molecular absorption. Nanoscale 2021, 13, 14497–14505. 10.1039/D1NR03913J. 34473173 PMC8412029

[ref29] Zhu Y.; Yang J.; Abad-Arredondo J.; Fernández-Domínguez A. I.; Garcia-Vidal F. J.; Natelson D. Electroluminescence as a probe of strong exciton-plasmon coupling in few-layer WSe2. Nano Lett. 2024, 24, 525–532. 10.1021/acs.nanolett.3c04684. 38109687

[ref30] Rider M. S.; Barnes W. L. Something from nothing: linking molecules with virtual light. Contemporary Physics 2021, 62, 217–232. 10.1080/00107514.2022.2101749.

[ref31] Vasista A. B.; Sharma D. K.; Kumar G. V. P. Digital Encyclopedia of Applied Physics*;* Wiley-VCH Verlag GmbH & Co. KGaA: Weinheim, Germany, 2019; pp 1–14.

[ref32] Tan W. J.; Thomas P. A.; Luxmoore I. J.; Barnes W. L. Single vs double anti-crossing in the strong coupling between surface plasmons and molecular excitons. J. Chem. Phys. 2021, 154, 024704 10.1063/5.0037864. 33445885

[ref33] Bellessa J.; Bonnand C.; Plenet J. C.; Mugnier J. Strong Coupling between Surface Plasmons and Excitons in an Organic Semiconductor. Phys. Rev. Lett. 2004, 93, 036404 10.1103/PhysRevLett.93.036404. 15323846

[ref34] Agranovich V. M.; Litinskaia M.; Lidzey D. G. Cavity polaritons in microcavities containing disordered organic semiconductors. Phys. Rev. B 2003, 67, 085311 10.1103/PhysRevB.67.085311.

[ref35] Schwartz T.; Hutchison J. A.; Léonard J.; Genet C.; Haacke S.; Ebbesen T. W. Polariton Dynamics under Strong Light–Molecule Coupling. ChemPhysChem 2013, 14, 125–131. 10.1002/cphc.201200734. 23233286

[ref36] Coles D. M.; Meijer A. J. H. M.; Tsoi W. C.; Charlton M. D. B.; Kim J.-S.; Lidzey D. G. A Characterization of the Raman Modes in a J-Aggregate-Forming Dye: A Comparison between Theory and Experiment. J. Phys. Chem. A 2010, 114, 11920–11927. 10.1021/jp107646p. 20945930

[ref37] Thomas P. A.; Tan W. J.; Fernandez H. A.; Barnes W. L. A New Signature for Strong Light–Matter Coupling Using Spectroscopic Ellipsometry. Nano Lett. 2020, 20, 6412–6419. 10.1021/acs.nanolett.0c01963. 32709208 PMC7608940

[ref38] Muccini M.; Murgia M.; Taliani C.; Esposti A. D.; Zamboni R. Optical properties and the photoluminescence quantum yield of organic molecular materials. Journal of Optics A: Pure and Applied Optics 2000, 2, 577 10.1088/1464-4258/2/6/313.

[ref39] Herrera F.; Spano F. C. Absorption and photoluminescence in organic cavity QED. Phys. Rev. A 2017, 95, 053867 10.1103/PhysRevA.95.053867.

[ref40] Herrera F.; Spano F. C. Dark Vibronic Polaritons and the Spectroscopy of Organic Microcavities. Phys. Rev. Lett. 2017, 118, 223601 10.1103/PhysRevLett.118.223601. 28621976

[ref41] Bhuyan R.; Lednev M.; Feist J.; Börjesson K. The Effect of the Relative Size of the Exciton Reservoir on Polariton Photophysics. Advanced Optical Materials 2024, 12, 2301383 10.1002/adom.202301383.

