# Supplementary material for: Molecular Strong Coupling and Cavity Finesse
Source: J Phys Chem Lett. 2024 Jul 15;15(29):7449–57. doi: 10.1021/acs.jpclett.4c00782 (PMC11284854; doi:10.1021/acs.jpclett.4c00782)
Supplement: Supplementary file 1 — jz4c00782_si_001.pdf [file jz4c00782_si_001.pdf]

Molecular Strong Coupling and Cavity Finesse (Supplementary Information)

Kishan S. Menghrajani,[*,†,§] Adarsh B. Vasista,[†,∥] Wai Jue Tan,[†] Philip A. Thomas,[†] Felipe Herrera,[‡,¶] and William L. Barnes[*,†]

†*Department of Physics and Astronomy, Stocker Road, University of Exeter, Devon EX4 4QL, United Kingdom*

‡*Department of Physics, Universidad de Santiago de Chile, Av. Victor Jara 3493, Santiago, Chile*

¶*Millennium Institute for Research in Optics, Concepción, Chile*

§*Current address: School of Physics and Astronomy, Monash University, Wellington Raid, Clayton, 3800, Victoria, Australia*

∥*Current address: Department of Physics, Indian Institute of Science Education and Research, Bhopal 462066, India*

E-mail: kishansmresearch@gmail.com; w.l.barnes@exeter.ac.uk

Contents

1 Strong coupling criterion **S2**

2 Optical Modes of different cavity structures **S3**

3 Optical Fourier set-up **S4**

4 Open Cavities **S5**

5 Half Cavities **S9**

6 Full Cavities **S12**

7 Estimating the Free Spectral Range and the cavity mode linewidth **S16**

8 Photoluminescence from different thin TDBC layers **S17**

9 Literature Values **S18**

10 Theory for Microcavity PL with Finite Finesse **S19**

11 Sample preparation **S23**

12 Reflectance Measurements **S23**

13 Coupled Oscillator Models **S23**

14 Transfer Matrix modelling **S25**

15 Technical Details re: TOC figure **S26**

1 Strong coupling criterion

A variety of criteria for the strong coupling regime are available in the literature.[1] The criterion is often expressed in words as the coupling rate exceeding the damping (molecular and cavity) rates. Whilst a formal discussion of the different criteria has some merit, in the present context the exact choice of which of the different criteria to use is not a key issue. What we attempt to do in the present work is to make a comparison between the criterion we chose ($\Omega_R = 2g_N > (K+\Gamma)/2$) and the emergence of PL associated with the lower polariton in our experiments.

2 Optical Modes of different cavity structures

It is worth clarifying what we mean here by ‘open’, ‘half’, and ‘full’ cavities. It is perhaps easiest to begin with the full cavity. The **full cavity** structure has a high reflectivity metallic mirror both above and below the molecular layer and is thus capable of supporting optical modes that are largely confined within the molecular layer (there will be a small degree of leakage of the fields through the metal mirrors). For the **half cavity** there is one high reflectivity (metallic) mirror under the molecular layer, the upper mirror is simply the low reflectivity interface between the molecular layer and air. Such structures may support well-confined optical modes when the in-plane wavevector of the mode is greater than the wavevector in air, i.e. in the regime of total internal reflection. For optical modes of lower wavevector the interface with the air provides only a weak reflection so that the optical field is no longer as well confined, such modes are often referred to as half-leaky modes. For the **open cavity** employed here we have a different situation. The lower interface between the molecular layer and the silicon substrate provides a modest degree of reflectivity whilst the interface between the molecular layer and air provides a somewhat lower reflectivity. Modes within the light line (in-plane wavevector less than that in air) are poorly confined to the molecular material, such modes are often referred to as leaky modes. For modes with an in-plane wavevector greater than that in air total internal reflection yields much more tightly confined modes.

For both the half and open cavities the situation can be rather complex when more than one mode is supported, such that one or more modes lie inside the light-line (and are thus not well-confined) and one or more lie beyond the light-line.[2]

3 Optical Fourier set-up

Figure S1 shows a schematic of the experimental setup. To measure angle resolved reflectance, a white light source was focused onto the sample using a 0.8 NA 100x objective lens and the reflected signal was collected using the same lens. Lens L4 and L5, together, project the back-focal plane of the objective lens onto either the spectrometer or the camera, depending on the position of the flip mirror FM2. Lens L6 was a flip-lens used to project the real plane to the spectrometer/camera. For Photoluminescence (PL) measurements, a beam-expanded diode-pumped 532 nm source was focused onto the sample using the objective lens, and the PL was collected in the back-scattering configuration. The laser line was then rejected using spectral edge filters placed after lens L5. For k∼0 excitation (normal incidence), we place a lens, L7, in the input path such that the laser was focused onto the back aperture of the objective lens, resulting in an approximate parallel beam with k∼0 at the sample plane.

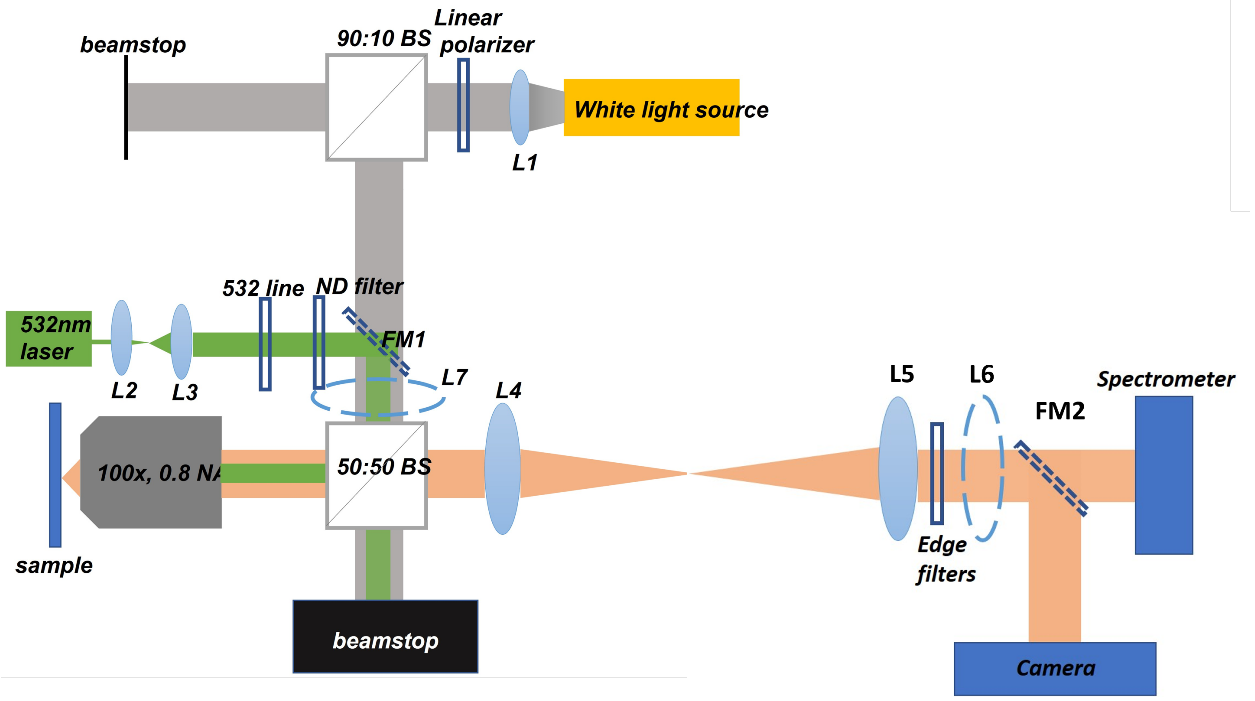

Figure S1: Schematic of the Fourier optical setup

4 Open Cavities

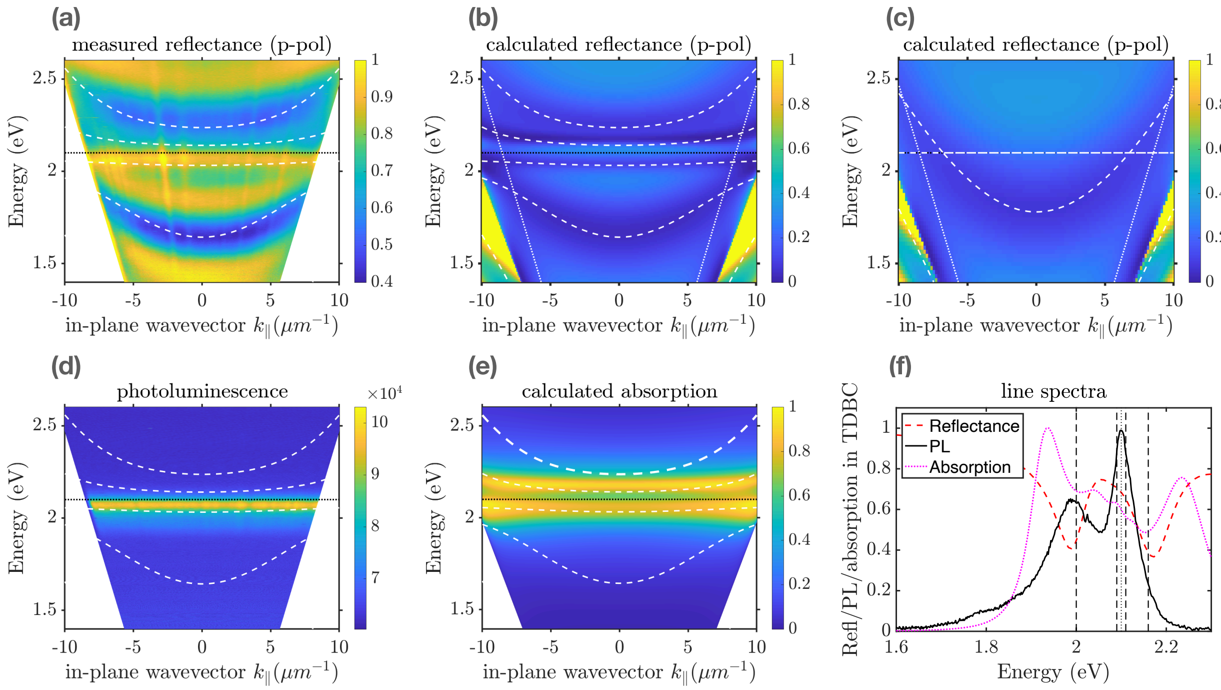

Figure S2: **Open cavity**, polymer thickness ∼340 nm. (a) Experimentally measured dispersion, based on p-polarised reflectance, indicated as a colour plot (see methods re: normalisation). (b) Calculated reflectance, the white dotted lines represent the limit of the collection numerical aperture used in the experiment (max angle = 55°). (c) As panel (b) except that in the calculation we have set the oscillator strength of the dye to zero. (d) Experimentally measured dispersion plot acquired from photoluminescence data. (e) Calculated absorption in the TDBC-doped layer. In panels (a-e) the polariton dispersion is shown, as determined from the coupled oscillator model described in the methods section. The polariton positions are shown as white dashed lines, and the molecular resonance (2.10 eV) by a black dotted line; just one cavity mode was used in the model (see panel (c)). (f) Normal incidence line spectra for calculated reflectance, measured PL and calculated absorption. The polariton positions (normal incidence) are indicated by black dashed lines.

In figure S2 we show, in the upper row, dispersion data based on reflectance for the open cavity, i.e. for a ∼340 nm TDBC-doped PVA layer on a silicon substrate. On the left (a) we show the measured reflectance, in the middle the calculated reflectance (b), and in (c) we again show the calculated reflectance, but this time we set the oscillator strength of the dye to zero. In the lower row we again show a dispersion plot, panel (d), this time based on the measured photoluminescence. In panel (e) we show the calculated absorption in the TDBC-doped PVA

layer, whilst in panel (f) we show normal incidence line spectra for the calculated reflectance, the measured photoluminescence and the calculated absorption in the TDBC. The white dashed lines in panels (a-e) indicate the positions of the polariton modes as determined from our coupled oscillator model (see methods), the polariton positions at normal incidence are indicated in panel (f) by black dashed lines. Further details are given in the figure caption.

Looking first at the reflectance, panels (a) and (b), the presence of modes of the open cavity system is indicated by regions of low reflectivity (blue). Also shown are the positions of the polaritons as determined by our coupled oscillator model (details in methods). We see that overall there is a reasonable match between the experimental data, the calculated data, and the coupled oscillator model (which here employed a single cavity resonance). The calculated dispersion for a 'no resonance' cavity and the 'no resonance' coupled oscillator model is shown in panel (c). Here 'no resonance' cavity and 'no resonance' coupled oscillator model refer to the oscillator strength being set to zero.

From the modelling we determine that in this case there are three photonic modes to consider, with de-tunings (relative to the molecular resonance) of -1.5, -0.32, and +0.85 eV, having beta values of 1.7, 1.6 and 1.9 respectively (β is a parameter that ensures the no-resonance cavity dispersion is correctly included in the coupled oscillator model). Note that these modes were determined by looking at data spanning a greater frequency range than shown here. The reduced oscillator strength is 0.14, and the splitting is $\Omega_R = 0.50 \pm 0.03$ eV. From figure S2(c) we find the mode width to be $K = 0.50 \pm 0.02$ eV.

In figure S3 we show, in the upper row, dispersion data based on reflectance for the open cavity, i.e. for a $\sim$1030 nm TDBC-doped PVA layer on a silicon substrate. The sample was similar to that used for the open cavity data shown in figure 2 of the main manuscript, the primary difference being that here the thickness of the cavity was 1030 nm rather than 340 nm used for the sample discussed in the main manuscript. On the left (a) we show the measured reflectance, in the middle the calculated reflectance (b), and in (c) we again show the calculated reflectance, but this time set the

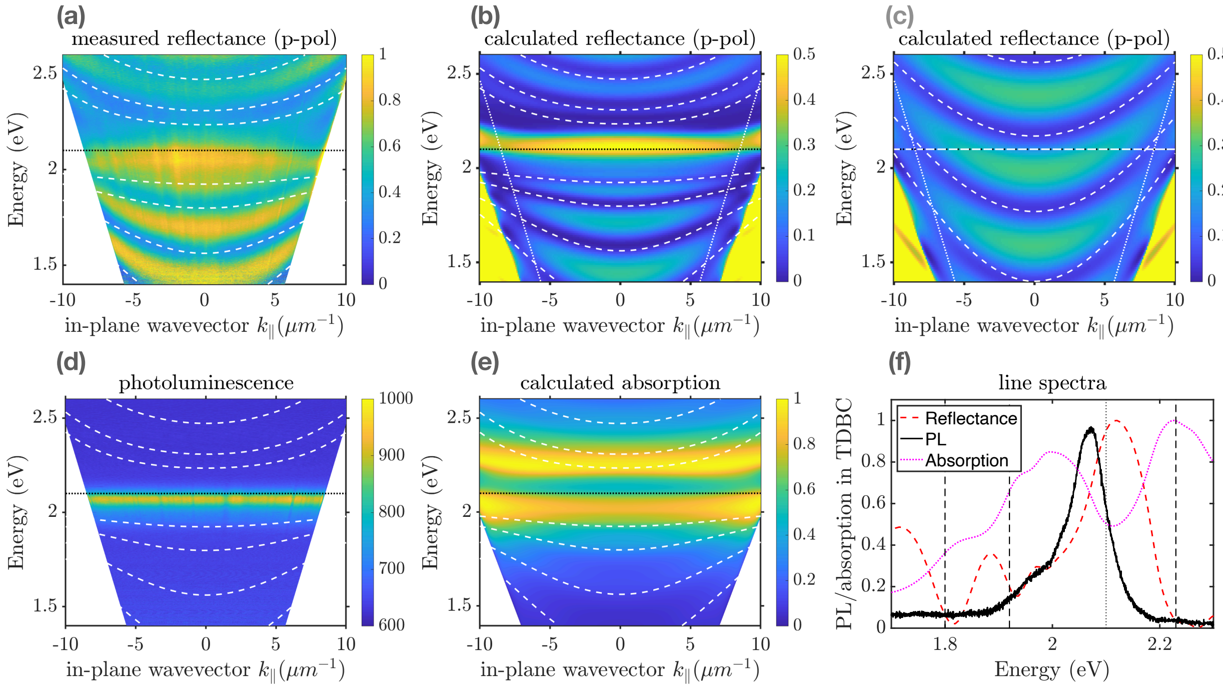

Figure S3: **Open Cavity (1030 nm)**. In panel (a) we show an experimentally measured dispersion, based on p-polarised reflectance, indicated as a colour plot (see methods re: normalisation). In panel (b) we show the calculated reflectance, the white dotted lines represent the limit of the collection numerical aperture used in the experiment. Panel (c) is the same as panel (b) except that in the calculation we have set the oscillator strength of the dye to zero. In panel (d) we show an experimentally measured dispersion plot acquired from photoluminescence data. In panel (e) we show the calculated absorption in the TDBC-doped layer. In panels (a-e) the polariton dispersion is shown, as determined from the coupled oscillator model described in the methods section. The polariton positions are shown as white dashed lines, and the molecular resonance (2.10 eV) by a black dotted line; three cavity modes were used in the model (see panel (c)), and the polymer thickness was ∼1030 nm. In panel (f) the polariton positions (normal incidence) are indicated by black dashed lines.

oscillator strength of the dye to zero. In the lower row we again show a dispersion plot, panel (d), this time based on the measured photoluminescence. In panel (e) we show the calculated absorption in the TDBC-doped PVA layer, whilst in panel (f) we show normal incidence line spectra for the measured reflectance, the measured photoluminescence and the calculated absorption in the TDBC. The white dashed lines in panels (a-e) indicate the positions of the polariton modes as determined from our coupled oscillator model (see methods), the polariton positions at normal incidence are indicated in panel (f) by black dashed lines. Further details are given in the figure caption.

Looking first at the reflectance, panels (a) and (b), the presence of modes of the open cavity system is indicated by regions of low reflectivity (blue). Also shown are the positions of the polaritons as determined by our coupled oscillator model (details in methods). We see that overall there is a reasonable match between the experimental data, the calculated data, and the coupled oscillator model (which here employed three cavity resonances). The calculated dispersion for a 'bare' cavity and the 'bare' coupled oscillator model is shown in panel (c). Here 'bare' refers to the oscillator strength in the Lorentz oscillator model being set to zero.

From the modelling we determine that in this case there four photonic modes to consider, with de-tunings of -0.7, -0.33, +0.07, and +0.46 eV, having beta values of 1.4, 1.2, 1.1 and 1.1 respectively. We determine that in this case the reduced oscillator strength is 0.25, the splitting is $\Omega = 0.67 \pm 0.04$ eV. Panel (d) shows the measured photoluminescence dispersion, we see that there is no sign of any influence of polaritons on the photoluminescence for this open cavity. Finally, to investigate the absorption we made further use of transfer matrix modelling, the results are shown in panel (e).

5 Half Cavities

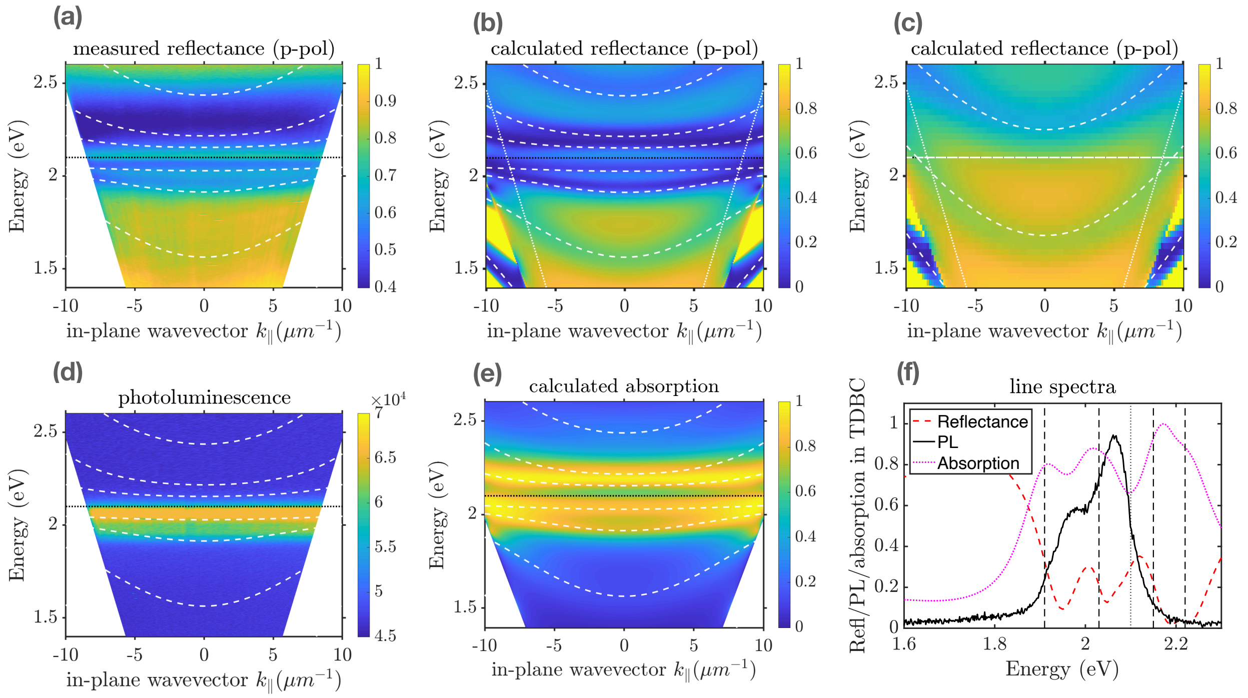

Figure S4: **Half cavity**, polymer thickness $\sim$ 600 nm. (a) Experimentally measured dispersion, based on p-polarised reflectance, indicated as a colour plot (see methods re: normalisation). (b) calculated reflectance, the white dotted lines represent the limit of the collection numerical aperture used in the experiment. (c) As panel (b) except that in the calculation we have set the oscillator strength of the dye to zero. (d) Experimentally measured dispersion plot acquired from photoluminescence data. (e) Calculated absorption in the TDBC-doped layer. In panels (a-e) the polariton dispersion is shown, as determined from the coupled oscillator model described in the methods section. The polariton positions are shown as white dashed lines, and the molecular resonance (2.10 eV) by a black dotted line; just one cavity mode was used in the model (see panel (c)). (f) Normal incidence line spectra for the calculated reflectance, the measured photoluminescence and the calculated absorption in the TDBC. The polariton positions (normal incidence) are indicated by black dashed lines.

Figure S6 shows data from a 600 nm TDBC-doped PVA layer on a gold-coated silicon substrate. Looking first at the reflectance, panels (a) and (b), the presence of the modes of the half cavity system are again indicated by regions of low reflectivity (blue). Also shown are the positions of the polaritons as determined by our coupled oscillator model, where we made use of two cavity modes. We note that overall the match between the experimental data, the calculated data,

and the coupled oscillator model are not as good as they were for the open cavity. The calculated dispersion for a 'no-resonance' cavity and the 'no-resonance' coupled oscillator model is shown in panel (c).

From the modelling we determine that in this case there are four photonic modes to consider, with de-tunings (relative to the molecular resonance of -1.1, -0.42, +0.15, and +0.80 eV, having beta values of 1.1, 1.1, 1.2 and 1.2 respectively. We determine that in this case the reduced oscillator strength is 0.14, and the splitting is $\Omega = 0.50 \pm 0.03$ eV. The width of the 'no-resonance' cavity modes in this case is $K = 0.25 \pm 0.01$ eV. Panel (d) shows the measured photoluminescence dispersion.

Figure S5 shows data from a 1630 nm TDBC-doped PVA layer on a gold-coated silicon substrate. Looking first at the reflectance, panels (a) and (b), the presence of modes of the half cavity system are again indicated by regions of low reflectivity (blue). Also shown are the positions of the polaritons as determined by our coupled oscillator model, where we made use of three cavity modes. We see that overall there is a reasonable match between the experimental data, the calculated data, and the coupled oscillator model. The calculated dispersion for a 'bare' cavity and the 'bare' coupled oscillator model is shown in panel (c).

From the modelling we determine that in this case there are five photonic modes to consider, with de-tunings (relative to the molecular resonance of -0.75, -0.50, -0.27, -0.02, and +0.25 eV, having beta values of 1.1, 1.1, 1.1, 1.1, and 1.0 respectively. We determine that in this case the reduced oscillator strength is 0.06, the splitting is $\Omega = 0.35 \pm 0.02$ eV. Panel (d) shows the measured photoluminescence dispersion. As for the 600 nm thick half cavity, things are now slightly more interesting than they were for the open cavity. The PL spectrum is somewhat different from the open cavity case, and there is perhaps some slight mapping of the PL onto the position of the lower polariton modes. Calculated data for the absorption in the TDBC are shown in panel (e). There is again a significant change in the absorption compared to that of the bulk (a single absorption peak),

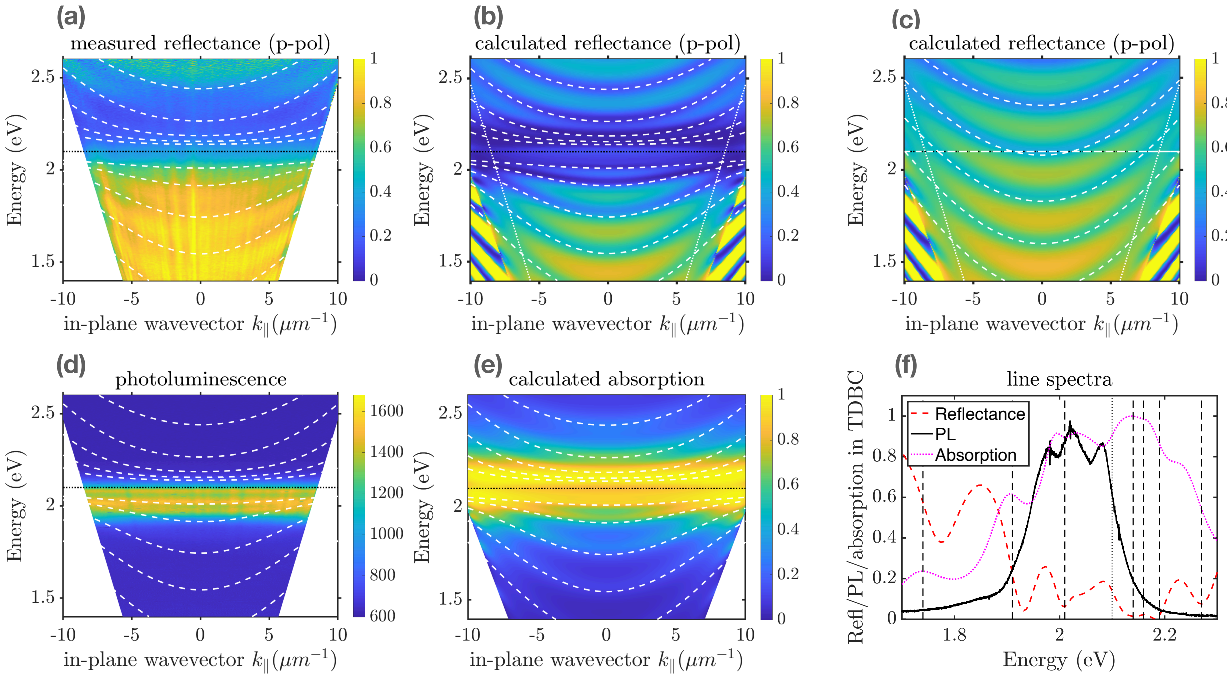

Figure S5: **Half Cavity (1630 nm)**. In panel (a) we show an experimentally measured dispersion, based on p-polarised reflectance, indicated as a colour plot. In panel (b) we show the calculated reflectance, the white dotted lines represent the limit of the collection numerical aperture used in the experiment. Panel (c) is the same as panel (b) except that in the calculation we have set the oscillator strength of the dye to zero. In panel (d) we show an experimentally measured dispersion plot acquired from photoluminescence data. In panel (e) we show the calculated absorption in the TDBC-doped layer. In panels (a-e) the polariton dispersion is shown, as determined from the coupled oscillator model described in the methods section. The polariton positions are shown as white dashed lines, and the molecular resonance (2.10 eV) by a black dotted line; three cavity modes were used in the model (see panel (c)), and the polymer thickness was $\sim$1630 nm. In panel (f) the polariton positions (normal incidence) are indicated by black dashed lines.

and further there is now an indication of the absorption tracking the polariton modes, at least in some very limited way.

6 Full Cavities

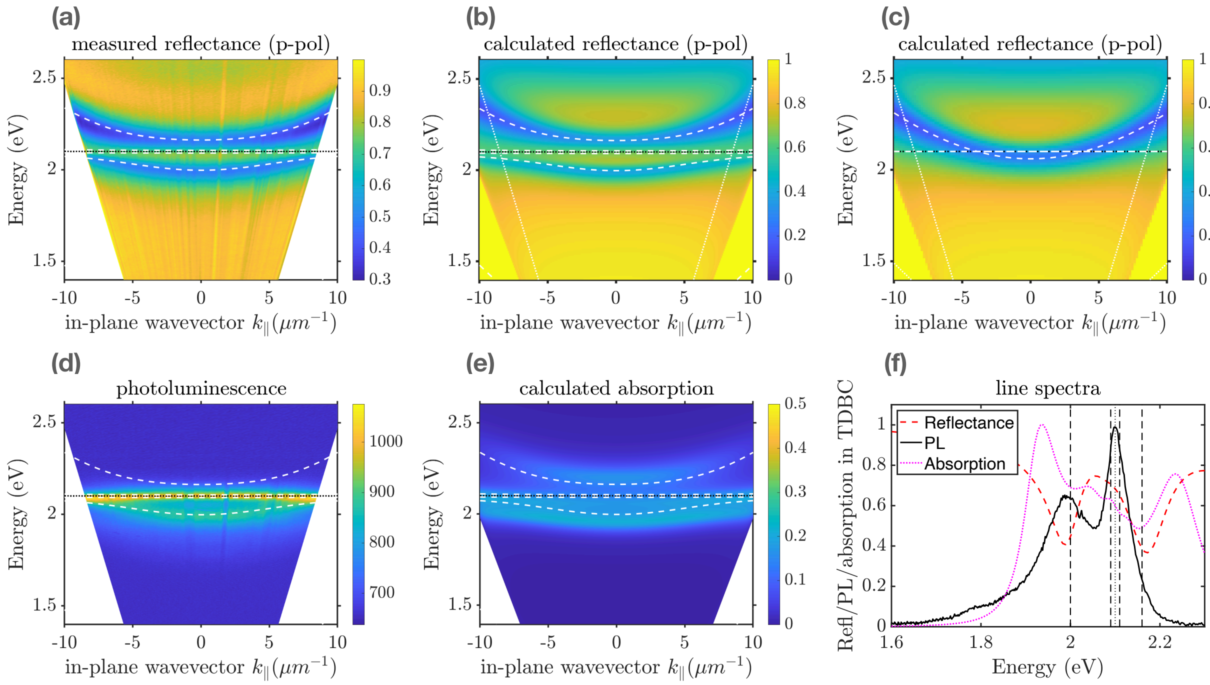

Figure S6: **Full cavity**, cavity thickness 330 nm. (a) Experimentally measured dispersion plot, based on p-polarised reflectance, indicated as a colour plot. (b) Calculated reflectance, the white dotted lines again represent the limit of the collection optics used in the experiment. (d) Photoluminescence. (e) Calculated absorption in the TDBC. In all plots the polariton dispersion is shown, as determined from the coupled oscillator model described in the methods section. The polariton positions are shown as white dashed lines, and the molecular resonance at 2.10 eV by a black dashed line; only one cavity mode was used in the model. (f) Normal incidence line spectra for the calculated reflectance, the measured photoluminescence and the calculated absorption in the TDBC. The polariton positions (normal incidence) are indicated by black dashed lines.

Again, as for the open and half cavities, in figure S6 we show the reflectance, panels (a) and (b), the presence of modes of the full cavity system are again indicated by regions of low reflectivity (blue). Also shown are the positions of the polaritons as determined by our coupled oscillator model, where we made use of just one cavity mode. We see that overall there is a reasonable match between the experimental data, the calculated data, and the coupled oscillator model. The calculated dispersion for a 'no-resonance' cavity and the 'no-resonance' coupled oscillator model is shown in panel (c). In panel (f) we show normal incidence line spectra for the calculated

reflectance, the measured photoluminescence and the calculated absorption in the TDBC. The calculated dispersion for a ‘no-resonance’ cavity and the ‘no-resonance’ coupled oscillator model are shown in panel (c).

From the modelling we determine that in this case there are three photonic modes to consider, with de-tunings (relative to the molecular resonance of -1.04, -0.04, and +0.72 eV, all having beta values of 0.65. The reduced oscillator strength is 0.018, the splitting is $\Omega_R = 0.16 \pm 0.01$ eV, and $\beta = 0.65$. The width of the ‘no-resonance’ cavity modes in this case is $K = 0.13 \pm 0.02$ eV.

For the two cavities that follow, the fabrication procedure was slightly different. We made use of a layer-by-layer deposition technique to place 4 monolayers of TDBC molecules (total 8 nm thick) inside these cavities, as follows. A 100 nm thick layer of PMMA was added on top of the first 40 nm gold mirror by spin coating, as to create part of the cavity. Then the TDBC film was deposited on top of this PMMA layer using a layer-by-layer approach.[3] Briefly, we used a cationic poly(diallyldimethylammonium chloride) (PDAC) solution as the polyelectrolyte binder for anionic TDBC J - aggregate solution. A typical deposition step consists of subsequent dipping the substrate inside a beaker of PDAC solution (20% by weight in water - diluted 1:1000) and TDBC solution in water (0.01M diluted 1:10) for 15 minutes each. The substrate was washed with DI water after each immersion and same steps were repeated to deposit multiple layers of PDAC/TDBC. To increase the adhesion we first coat one layer of anionic polystyrene sulfonate (PSS) using the above mentioned process and continue with PDAC - TDBC. Finally the TDBC layer was protected by depositing a layer of PDAC molecules. The superstrate was then prepared by spinning a second layer of PMMA and finally the top mirror was prepared by thermally evaporating a further 40 nm of gold.

We discuss the results for both the 355 nm and 400 nm cavities at the same time since they are very similar. Looking first at the reflectance, panels (a) and (b), the presence of modes of the full cavity system are again indicated by regions of low reflectivity (blue). Also shown are the positions

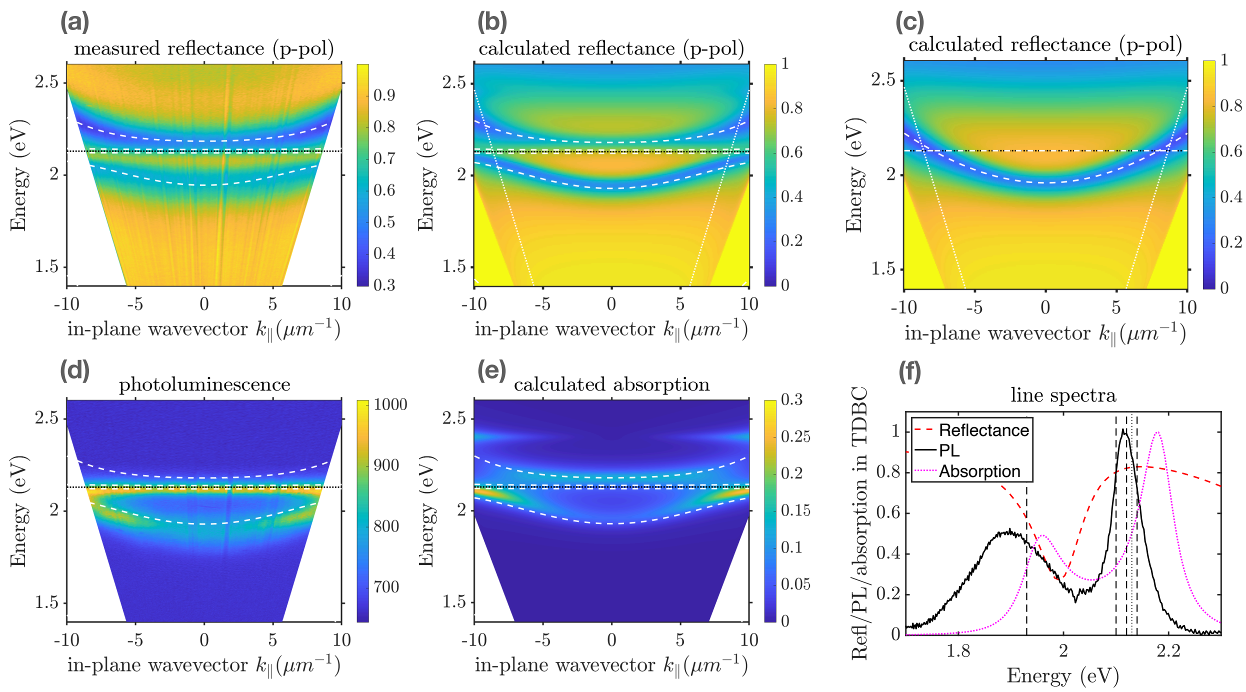

Figure S7: **Full Cavity (355 nm)**. In panel (a) we show an experimentally measured dispersion, based on p-polarised reflectance, indicated as a colour plot. In panel (b) we show the calculated reflectance, the white dotted lines represent the limit of the collection numerical aperture used in the experiment. Panel (c) is the same as panel (b) except that in the calculation we have set the oscillator strength of the dye to zero. In panel (d) we show an experimentally measured dispersion plot acquired from photoluminescence data. In panel (e) we show the calculated absorption in the TDBC-doped layer. In panels (a-e) the polariton dispersion is shown, as determined from the coupled oscillator model described in the methods section. The polariton positions are shown as white dashed lines, and the molecular resonance (2.13 eV) by a black dotted line; just one cavity mode was used in the model (see panel (c)), and the cavity thickness was ∼355 nm. In panel (f) the polariton positions (normal incidence) are indicated by black dashed lines.

of the polaritons as determined by our coupled oscillator model, where we made use of one cavity mode (see panel (c)). We see that overall there is a reasonable match between the experimental data, the calculated data, and the coupled oscillator model. The calculated dispersion for a 'bare' cavity and the 'bare' coupled oscillator model is shown in panel (c).

From the modelling we determine that in both cases there are three photonic modes to consider: for the 355 nm cavity, the de-tunings are -1.13, -0.15, and +1.06 eV, all having beta values of 0.65. The reduced oscillator strength is 0.63, and the splitting is $\Omega_R = 0.20 \pm 0.02$ eV; for the 400 nm cavity, the de-tunings are -1.23, -0.30, and +0.40 eV, all having beta values of 0.7. The reduced

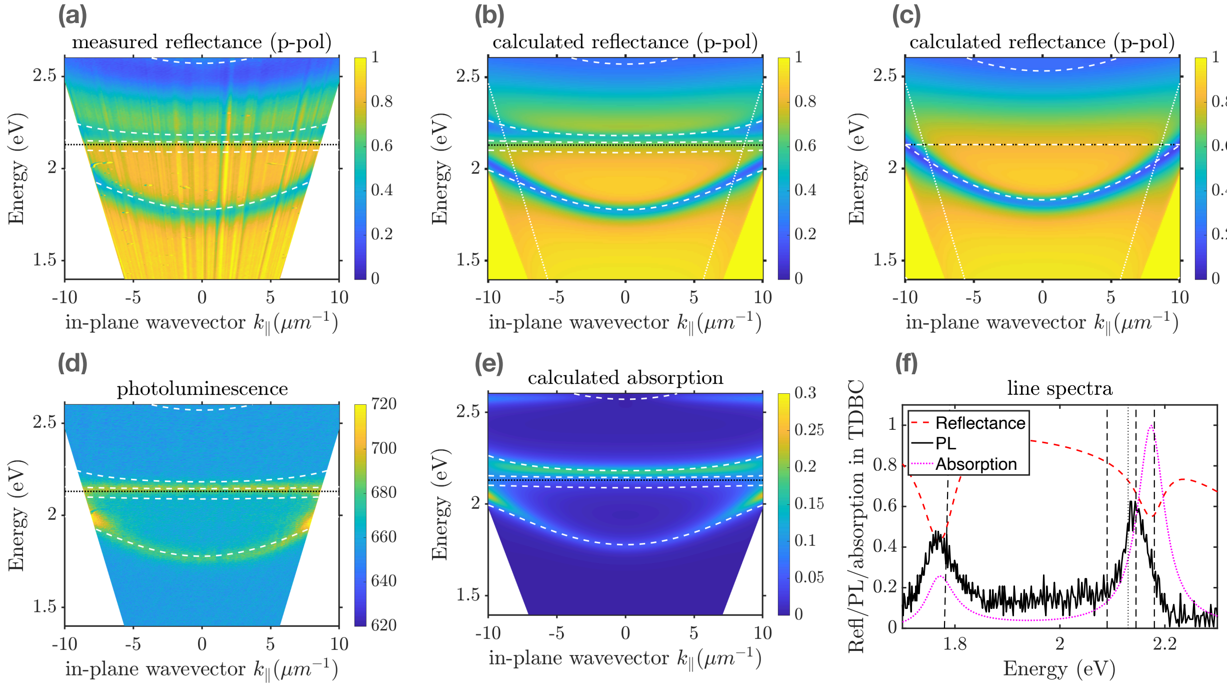

Figure S8: **Full Cavity (400 nm)**. In panel (a) we show an experimentally measured dispersion, based on p-polarised reflectance, indicated as a colour plot. In panel (b) we show the calculated reflectance, the white dotted lines represent the limit of the collection numerical aperture used in the experiment. Panel (c) is the same as panel (b) except that in the calculation we have set the oscillator strength of the dye to zero. In panel (d) we show an experimentally measured dispersion plot acquired from photoluminescence data. In panel (e) we show the calculated absorption in the TDBC-doped layer. In panels (a-e) the polariton dispersion is shown, as determined from the coupled oscillator model described in the methods section. The polariton positions are shown as white dashed lines, and the molecular resonance (2.13 eV) by a black dotted line; just one cavity mode was used in the model (see panel (c)), and the cavity thickness was ∼400 nm. In panel (f) the polariton positions (normal incidence) are indicated by black dashed lines.

oscillator strength is 1.08, and the splitting is $\Omega_R = 0.27 \pm 0.02$ eV.

Panel (d) in figures S7 and S8 both show the measured photoluminescence dispersion, the PL spectrum is very clearly different from the open and half cavity cases, the PL nicely mapping onto the position of the lower polariton mode. Calculated data for the absorption in the TDBC are shown in panel (e). There is again a very significant change in the absorption compared to that of the bulk (a single absorption peak), the absorption now tracking the polariton modes.

7 Estimating the Free Spectral Range and the cavity mode linewidth

The bare cavity mode linewidth was estimated from the calculated reflectance data with the oscillators turned off, e.g. panel C of figure S2 etc.. The estimate was based on the FWHM at an in-plane wavevector that corresponded to the crossing point with the excitonic resonance.

The Free Spectral Range was estimated from the average of the frequency difference to the next nearest modes, i.e. one mode higher in frequency, one mode lower in frequency.

8 Photoluminescence from different thin TDBC layers

As a comparison for the PL data we recorded from the different cavities, in figure S9 we show the PL spectra of thin films of TDBC-doped material.

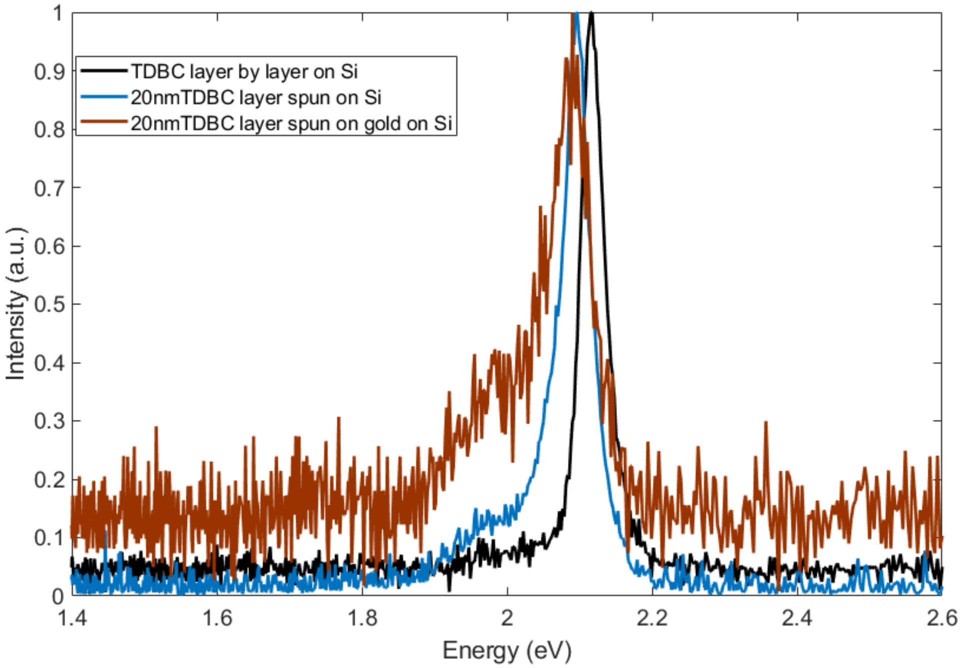

Figure S9: **Photoluminescence from thin TDBC layers**. Three spectra are shown: PL from TDBC produced by the layer-by-layer technique, deposited on Si; PL from TDBC-doped PVA on Si; PL from TDBC-doped PVA on Au coated Si.

Note that the PL for TDBC-doped PVA peaks at a lower energy (∼2.10 eV) than the TDBC produced using the layer-by-layer technique (∼2.13 eV). Note also that for the TDBC-doped PVA on Au coated Si, the low energy shoulder of the TDBC PL is enhanced in relative strength. This is consistent with a recent observation based on squarine dyes.[4] We further note that by using ellipsometry[5] we determined that the resonance energy for the layer-by-layer TDBC film is at 2.14eV, i.e. to within experimental error it is the same as the 2.13eV of the PL reported here.

9 Literature Values

Data used for the numbered points in figure 3 are as follows:

1) Wersall *et al.*[6] The numbers we extracted from this work for inclusion in the present manuscript are: $\Omega_R = 0.23 \pm 0.03$ eV, FSR = 2.1±0.1 eV (we assume the nano-prism only supports one relevant plasmonic mode), $K = 0.21 \pm 0.03$ eV, $\Gamma = 0.10 \pm 0.01$ eV. These lead to a value for the finesse of $\mathscr{F} = 10 \pm 2$, and a value for $2\Omega_R/(\Gamma + K)$ of 1.5 ±0.2.

2) Thomas *et al.*[7] The numbers we extracted from this work for inclusion in the present manuscript are: $\Omega_R = 0.59 \pm 0.03$ eV, FSR = 1.2±0.1 eV, $K = 0.46 \pm 0.01$ eV (note this is a more recent estimate than the originally published data), $\Gamma = 0.36 \pm 0.01$ eV. These lead to a value for the finesse of $\mathscr{F} = 2.6 \pm 0.4$, and a value for $2\Omega_R/(\Gamma + K)$ of 1.4 ±0.2.

3) Vasista *et al.*[8] The numbers we extracted from this work for inclusion in the present manuscript are: $\Omega_R = 0.094 \pm 0.01$ eV, FSR = 0.10±0.01 eV, $K = 0.03 \pm 0.005$ eV, $\Gamma = 0.11 \pm 0.01$ eV. These lead to a value for the finesse of $\mathscr{F} = 3.3 \pm 1.1$, and a value for $2\Omega_R/(\Gamma + K)$ of 1.4 ±0.2.

4) Thomas *et al.*[9] The numbers we extracted from this work for inclusion in the present manuscript are: $\Omega_R = 70 \pm 5$ cm^{-1}, FSR = 420±10 cm^{-1} $K = 30 \pm 5$ cm^{-1}, $\Gamma = 30 \pm 2$ cm^{-1}. These lead to a value for the finesse of $\mathscr{F} = 14.0 \pm 0.4$, and a value for $2\Omega_R/(\Gamma + K)$ of 2.3 ±0.1.

5) Ahn *et al.*[10] The numbers we extracted from this work for inclusion in the present manuscript are: $\Omega_R = 73 \pm 6$ cm^{-1}, FSR = 480±10 cm^{-1} $\omega_0 = 2260$ cm^{-1}, $K = 28 \pm 6$ cm^{-1}, $\Gamma = 47 \pm 1$ cm^{-1}. These lead to a value for the finesse of $\mathscr{F} = 11.8 \pm 0.3$, and a value for $2\Omega_R/(\Gamma + K)$ of 2.6 ±0.2.

10 Theory for Microcavity PL with Finite Finesse

The derivation of Eq. (3) for the photoluminescence (PL) spectrum of the lower polariton is given, starting from a microscopic quantum model. The stationary photoluminescence spectrum $S(\omega)$ of a microcavity is given by the two-time autocorrelation function of the q-th cavity mode [11]

$$S_q(\omega) = \int_0^\infty d\tau \langle \hat{a}_q^\dagger(\tau)\hat{a}_q(0)\rangle e^{i\omega\tau}, \tag{1}$$

where $\hat{a}_q$ is the annihilation operator of the mode. Using the quantum regression formula, Eq. (1) can be evaluated analytically in the polariton basis in the approximation that no quantum jumps occur, to give[11]

$$S_q(\omega) = \sum_{ij} \rho_j \left|\left\langle \varepsilon_i|\hat{a}_q|\varepsilon_j\right\rangle\right|^2 \frac{(\Gamma_j/2)}{(\omega-\omega_{ji})^2 + (\Gamma_j/2)^2}, \tag{2}$$

where ρ_j is the stationary population of the coupled system eigenstate $|\varepsilon_j\rangle$, Γ_j is the decay rate of $|\varepsilon_j\rangle$, and $\omega_{ji} = \varepsilon_j - \varepsilon_i$ is the frequency of the transition $|\varepsilon_j\rangle \to |\varepsilon_i\rangle$, with $\varepsilon_j > \varepsilon_i$ being the state energies. Considering only the the ground and first excitation manifolds, which determine the linear response of the system, Eq. (2) can be written as

$$S_q(\omega) = \pi \sum_j \rho_j \left|\langle G|\hat{a}_q|\varepsilon_j\rangle\right|^2 L_j(\omega) \tag{3}$$

where $|G\rangle = |g_1, g_2, \ldots, g_N\rangle\,|\{0_q\}\rangle$ is the global ground state with all dipoles in a local ground state $|g_n\rangle$ and all cavity modes in the vacuum state. $L_j(\omega)$ is a normalized Lorentzian lineshape function centered at ω_j with bandwidth Γ_j (FWHM), associated with the transition $G \to \varepsilon_j$.

As shown by Herrera and Spano,[11] solving for the eigenstates and energies of all the polaritonic and dark states of the coupled light-matter system in the single excitation manifold, enables the direct evaluation of Eq. (3), up to an undetermined excited population parameter ρ_j. Instead of assuming an arbitrary state-independent value $\rho_j \sim 1/N$,[11] we derive an analytical expression for ρ_j following the observation that ultraviolet pumping of material dipoles followed by ultrafast

energy relaxation down to the lowest singlet (S_1) excited state is equivalent to the direct incoherent excitation of local molecular dipoles with jump operator $\hat{L}_j = \sqrt{W}\hat{\sigma}_{+n}$, where $\hat{\sigma}_{+n}$ creates an excitation in the n-th molecular dipole and W is the effective incoherent pumping rate. The proposed equivalence can be justified using standard adiabatic elimination techniques.[12]

The quantum master equation that describes incoherent dipole pumping and cavity decay can be written in Lindblad form as

$$\frac{d\hat{\rho}}{dt} = -i[\hat{H},\hat{\rho}] + \sum_n W_n \left(\hat{\sigma}_{+n}\hat{\rho}\hat{\sigma}_{-n} - \frac{1}{2}\{\hat{\sigma}_{-n}\hat{\sigma}_{+n},\hat{\rho}\}\right) + \sum_q \kappa_q \left(\hat{a}_q\hat{\rho}\hat{a}_q^\dagger - \frac{1}{2}\left\{\hat{a}_q^\dagger\hat{a}_q\hat{\rho}\right\}\right), \quad (4)$$

where $\hat{H}$ is the coupled light-matter Hamiltonian representing N two-level dipoles in a multimode microcavity, W_n is the local excitation rate of the n-th dipole and κ_q is the decay rate of the q-th cavity mode. $\{\hat{A},\hat{B}\}$ denotes an anticommutator. For long times $t \gg 1/W_n$, we expect the incoherently driven system to evolve into mixed state $\hat{\rho} = \sum_j \rho_j |\varepsilon_j\rangle\langle\varepsilon_j|$, with state population $0 < \rho_j < 1$. Equations of motion for the population of coupled eigenstates $\rho_j^{(1)}$ in the first-excitation manifold are obtained directly from Eq. (4) in the mixed-state ansatz $\hat{\rho} = (1-\chi_W)|G\rangle\langle G| + \sum_j \rho_j^{(1)} |\varepsilon_j\rangle\langle\varepsilon_j|$, giving

$$\frac{d}{dt}\rho_j^{(1)} = W\sum_n \left|\langle\varepsilon_j|\hat{\sigma}_n^+|G\rangle\right|^2 (1-\chi_W) - W\sum_n \langle\varepsilon_j|\sigma_n^-\sigma_n^+|\varepsilon_j\rangle\rho_j^{(1)} - \kappa\sum_q \langle\varepsilon_j|\hat{a}_q^+\hat{a}_q|\varepsilon_j\rangle\rho_j^{(1)}, \quad (5)$$

where homogeneous pumping and mode-independent bandwidth conditions were assumed ($W_n = W$ and $\kappa_q = \kappa$). The ground depletion parameter $\chi_W = \sum_j \rho_j^{(1)}$ is introduced for completeness, but can be neglected for the weak pumping conditions $W \ll \kappa$ relevant to this work. Although the second-moment $\langle\varepsilon_j|\sigma_n^-\sigma_n^+|\varepsilon_j\rangle$ virtually couples the state with the second-excitation manifold, it is kept in the Eq. (5) because it does not involve double excitation of the same dipole.

The steady state solution of Eq. (5) with $\chi_W \to 0$ is

$$\rho_{jss}^{(1)} = \frac{W\sum_n \left|\langle\varepsilon_j|\hat{\sigma}_n^+|G\rangle\right|^2}{W\langle\varepsilon_j|\sum_n \sigma_n^-\sigma_n^+|\varepsilon_j\rangle + \kappa\langle\varepsilon_j|\sum_q \hat{a}_q^+\hat{a}_q|\varepsilon_j\rangle}, \quad (6)$$

which can be evaluated explicitly within the single-excitation wavefunction ansatz

$$|\varepsilon_j\rangle = \sum_n c_{i\varepsilon_j}|g_1,\ldots,e_n,\ldots g_N\rangle|\{0_q\}\rangle + \sum_q c_{q\varepsilon_j}|G\rangle|1_q\rangle, \tag{7}$$

to give

$$\rho^{(1)}_{jss} = \frac{WX_j^T}{W(N-1)X_j^T + (NW+\kappa)C_j^T}, \tag{8}$$

where $X_j^T = \sum_n |c_{n\varepsilon_j}|^2$ is the total exciton fraction of the j-th coupled eigenstate $|\varepsilon_j\rangle$ and $C_j^T = \sum_q |c_{q\varepsilon_j}|^2$ is the total photon fraction. Combining Eq. (8) with (2), the analytical expression for the PL spectrum in the limit $N \to \infty$, with NW finite, becomes

$$S_q(\omega) = \pi W \sum_j \frac{X_j^T C_{jq}}{NWX_j^T + (NW+\kappa)C_j^T} L_j(\omega), \tag{9}$$

which is Eq. (3) in the main text, were the cavity decay rate is expressed in terms of bandwidth $K = 2\kappa$ (FWHM). In the limit where photon loss dominates over the excitation rate, i.e., $\kappa \gg NW$ and the free spectral range is infinite, $C_j^T = C_j^q$, the cavity PL spectrum reduces to $S_T(\omega) \approx \pi W \sum_j X_j^T L_j(\omega)$, i.e., is fully determined by the exciton content at particular system eigenfrequencies ω_j.

Figure S10 shows a spectrum of the terms that contribute to the PL signal in Eq. (9): total exciton content X_j^T, total photon content C_j^T, the product $X_j^T C_{jq}$, and the denominator $NWX_j^T + (NW+\kappa)C_j^T$, for each light-matter eigenstate in a model system composed of an ensemble of $N = 150$ excitonic dipoles with Gaussian disorder centered at $\omega_e = 2.1$ eV ($\sigma = 0.05$ eV) interacting with a two-mode cavity with mode frequencies $\omega_{q-1} = 1.8$ eV and $\omega_q = 2.1$ eV, both having equal bandwidth $\kappa = 0.1$ eV. Each molecular dipole couples to both cavity modes with single-molecule Rabi frequency $g = 0.2/\sqrt{N}$ eV. We use $NW = 0.0001$ and average over 650 disorder configurations, consistent with the results in Fig. 7 of the main text.

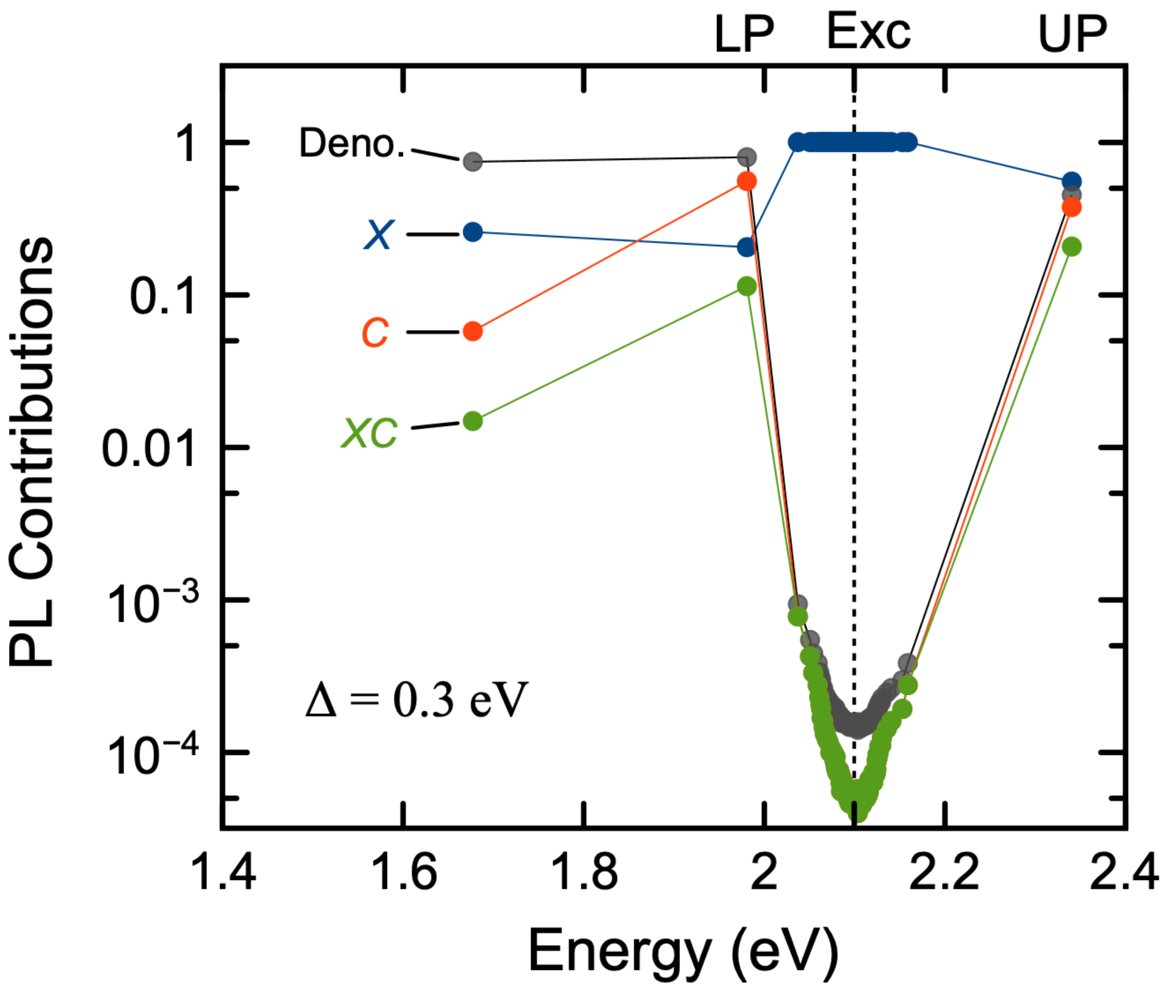

Figure S10: **Spectrum of contributions to the expression for PL**, including total exciton content X [X_j^T in Eq. (9)], total photon content C (C_j^T), light-matter product XC ($X_j^T C_{jq}$), and denominator $NWX_j^T + (NW+\kappa)C_j^T$, for a two-mode cavity with (low) finesse $\Delta = 0.3$ eV that couples to a Gaussian distribution of exciton transitions (Exc) centered at 2.1 eV (σ =0.05 eV, $\kappa = K/2 = 0.1$ eV). Contributions are averaged over 650 disorder configurations.

11 Sample preparation

Solution preparation: Poly Vinyl Acetate (PVA, molar weight 450 000) was used as a host matrix for TDBC. PVA was dissolved in water. TDBC was then dissolved in the PVA-water solution. TDBC/PVA films were deposited on a silicon wafer by spin-coating. This produced film thicknesses over the range 300-1700 nm.

For the open-cavity structures, a TDBC/PVA solution was spun on top of a silicon substrate. For the half-cavity a thin gold layer of 30 nm was deposited on top of silicon substrate by thermal evaporation. Later, TDBC/PVA was spun on top of gold thin film. For the full-cavity, the lower mirror was prepared by thermally evaporating 40 nm of gold onto a silicon substrate. Then, following deposition of the TDBC/PVA layer, a second 40 nm gold mirror was deposited, again by thermal evaporation.

12 Reflectance Measurements

Whilst substrate-only spectra for the reflectance should provide a means to normalise the reflectance data, in our samples a non-trivial level of scattering meant that the normalisation process was not as effective as hoped. Accordingly the reflectance spectra were scaled to provide a better match with calculated data.

13 Coupled Oscillator Models

We made use of a standard coupled oscillator model to help us interpret our data.[13] The number of cavity resonances included in each model depended on the details of the sample (thickness etc.). For a molecular resonance at a frequency (angular) of ω_{mol} interacting with several cavity resonances (whose energy depend on in-plane wavevector, i.e. $\omega_{\mathrm{cav}}(k_{\parallel})$), the coupled oscillator model allows the dispersion of the new hybrid (polariton) modes to be determined. Since all of our samples supported more than one cavity modes we made use of a 2N multi-photonic-mode

coupled oscillator model[13,14] to find the polariton frequencies by solving for those frequencies ω for which the determinant of the coupling matrix is zero, i.e. $|\mathbf{M}| = 0$. Although this choice (2N) is not universal, many authors preferring an N+1 coupled oscillator model, we find it to be more universally applicable. As an example, the coupling matrix for one molecular resonance interacting with two cavity (photonic) modes is given by,

$$\mathbf{M} = \begin{pmatrix} \omega_{\mathrm{mol}} - \omega & \Omega_R/2 & 0 & 0 \\ \Omega_R/2 & \omega_1(k_{||}) - \omega & 0 & 0 \\ 0 & 0 & \omega_{\mathrm{mol}} - \omega & \Omega_R/2 \\ 0 & 0 & \Omega_R/2 & \omega_2(k_{||}) - \omega \end{pmatrix} \tag{10}$$

where Ω_R is the extent of the interaction (assumed to be the same for all modes), and is equal to the frequency splitting (anti-crossing) at the value of $k_{||}$ where the uncoupled resonances cross. For the nth cavity resonance we described the $k_{||}$ dependence, $\omega_n(k_{||})$, as,

$$\omega_n = \frac{\omega_{n\,k_0=0}}{k_0}\sqrt{{k_0}^2 + \beta_n {k_{||}}^2/\varepsilon_b}, \tag{11}$$

where $\omega_{n\,k_0=0}$ is the angular frequency of the nth resonance at $k_{||} = 0$. The factor β_n was used to match the dispersion of the bare cavity modes with the calculated bare reflectance (needed because the coupled oscillator model ignores the angle-dependant phase change on reflection from an interface). We chose to work with the simplest model that we could, and therefore assumed no damping of the modes. The values of the $\omega_{n\,k_0=0}$, of Ω_R and of β_n were determined by simultaneously trying to match the eigenvalues to the calculated and measured reflection data. The values for the Rabi splitting we report here are based on the parameters used in the coupled oscillator model that best match our data.

14 Transfer Matrix modelling

We made use of a standard multi-layer optics approach to calculations of the reflectance and absorption,[15–17] and assumed all media were isotropic. The reflectance was calculated by taking the square of the modulus of the amplitude reflection coefficient. For the permittivities, we used the following:

Silicon substrate: we took the permittivity to be 16.0 + 3.0i.

Gold films: we used a Drude-Lorentz model for the frequency/energy dependant permittivity of gold, $\varepsilon_{\text{Au}}(\omega)$, given by,[18]

$$\varepsilon_{\text{Au}}(\omega) = \frac{f_d\,\omega_p^2}{\omega^2 + i\gamma_d\omega} + \sum_j \frac{f_j\omega_p^2}{\omega_j^2 - \omega^2 - i\gamma_j\omega}, \tag{12}$$

where the plasma frequency ω_p was 9.03 eV, the Drude damping rate γ_d was 0.053 eV, and the strength, f_d was 0.76. The Lorentz oscillator parameters we used are,

Table 1: Lorentz oscillator model parameters for gold films, as determined from ellipsometry

j	f_j	$\omega_j(eV)$	$\gamma_j(eV)$
1	0.024	0.451	0.241
2	0.010	0.830	0.345
3	0.071	2.969	0.870
4	0.601	4.304	2.494
5	4.385	13.32	2.214

TDBC J-aggregates: we used a Lorentz model, with the permittivity $\varepsilon_{\text{TDBC}}(E)$ given by,

$$\varepsilon_{\text{TDBC}}(\omega) = \varepsilon_b + \frac{f\,\omega_0^2}{\omega_0^2 - \omega^2 - i\gamma\omega}, \tag{13}$$

15 Technical Details re: TOC figure

The two spectra show the calculated transmission spectra for two of the cavities we investigated, one of low finesse (~ 2) and one of higher finesse (~ 8). Note that the spectrum for the lower finesse cavity shows modes that overlap.

The two curves shown in the TOC figure are the zero-oscillator normal incidence transmittance (calculated) for the following two cavity structures.

We show the calculated transmittance (empty cavity, i.e. zero oscillator strength) for two of our samples, the 600 nm half cavity (dotted line) and the 330 nm full cavity (full line).

Half cavity (600 nm), dotted red line.

Full cavity (330 nm), full blue line.

References

(1) Rider, M. S.; Barnes, W. L. Something from nothing: linking molecules with virtual light. *Contemporary Physics* **2021**, *62*, 217–232.

(2) Menghrajani, K. S.; Barnes, W. L. Strong coupling beyond the light-line. *ACS Photonics* **2020**, *7*, 2448–2459.

(3) Bradley, M. S.; Tischler, J. R.; Bulović, V. Layer-by-Layer J-Aggregate Thin Films with a Peak Absorption Constant of 106 cm–1. *Adv. Mater.* **2005**, *17*, 1881–1886.

(4) Quenzel, T.; Timmer, D.; Gittinger, M.; Zablocki, J.; Zheng, F.; Schiek, M.; Lützen, A.; Frauenheim, T.; Tretiak, S.; Silies, M.; Zhong, J.-H.; De Sio, A.; Lienau, C. Plasmon-Enhanced Exciton Delocalization in Squaraine-Type Molecular Aggregates. *ACS Nano* **2022**, *16*, 4693–4704.

(5) Rider, M. S.; Johnson, E. C.; Bates, D.; Wardley, W. P.; Gordon, R. H.; Oliver, R. D. J.; Armes, S. P.; Leggett, G. J.; Barnes, W. L. Strong coupling in molecular systems: Strong

coupling in molecular systems: a simple predictor employing routine optical measurements. *Nanophotonics* **2024**,

(6) Wersäll, M.; Cuadra, J.; Antosiewicz, T. J.; Balci, S.; Shegai, T. Observation of Mode Splitting in Photoluminescence of Individual Plasmonic Nanoparticles Strongly Coupled to Molecular Excitons. *Nano Letters* **2016**, *17*, 551–558.

(7) Thomas, P. A.; Menghrajani, K. S.; Barnes, W. L. Cavity-Free Ultrastrong Light-Matter Coupling. *The Journal of Physical Chemistry Letters* **2021**, *12*, 6914–6918.

(8) Vasista, A.; Barnes, W. L. Molecular monolayer strong coupling in dielectric soft microcavities. *Nano Letters* **2020**, *20*, 1766–1773.

(9) Thomas, A.; Lethuillier-Karl, L.; Nagarajan, K.; Vergauwe, R. M. A.; George, J.; Chervy, T.; Shalabney, A.; Devaux, E.; Genet, C.; Moran, J.; Ebbesen, T. W. Tilting a ground-state reactivity landscape by vibrational strong coupling. *Science* **2019**, *363*, 615–619.

(10) Ahn, W.; Triana, J. F.; Recabal, F.; Herrera, F.; Simpkins, B. S. Modification of ground-state chemical reactivity via light-matter coherence in infrared cavities. *Science* **2023**, *380*, 1165–1168.

(11) Herrera, F.; Spano, F. C. Absorption and photoluminescence in organic cavity QED. *Phys. Rev. A* **2017**, *95*, 053867.

(12) Zhu, B.; Schachenmayer, J.; Xu, M.; Herrera, F.; Restrepo, J. G.; Holland, M. J.; Rey, A. M. Synchronization of interacting quantum dipoles. *New Journal of Physics* **2015**, *17*, 083063.

(13) Richter, S.; Michalsky, T.; Fricke, L.; Sturm, C.; Frank, H.; Grundmann, M.; Schmidt-Grund, R. Maxwell consideration of polaritonic quasi-particle Hamiltonians in multi-level systems. *Applied Physics Letters* **2015**, *107*, 231104.

(14) Balasubrahmaniyam, M.; Genet, C.; Schwartz, T. Couling and decoupling of polaritonic states in multimode cavities. *Physical Review B* **2021**, *103*, L241407.

(15) Smith, L. H.; Taylor, M. C.; Hooper, I. R.; Barnes, W. L. Field profiles of coupled surface plasmon-polaritons. *Journal of Modern Optics* **2008**, *55*, 2929–2943.

(16) Novotny, L.; Hecht, B. *Principles of nano-optics*, 1st ed.; Cambridge University Press: Cambridge, UK, 2006.

(17) Tan, W.-J. Anti-crossing and strong coupling in interactions of molecules with confined light fields. PhD, Physics and Astronomy, University of Exeter, 2023.

(18) Rakić, A. D.; Djurišić, A. B.; Elazar, J. M.; Majewski, M. L. Optical properties of metallic films for vertical-cavity optoelectronic devices. *Appl. Opt.* **1998**, *37*, 5271–5283.
